# Borderline personality disorder and other psychiatric, somatic, and behavioral conditions: a nationwide family study

**DOI:** 10.1038/s41398-026-04001-w

**Published:** 2026-04-10

**Authors:** Alisha S. M. Hall, Katherine L. Musliner, Jean-Christophe P. Debost, Bjarni J. Vilhjálmsson, Zheng Chang, Brian M. D’Onofrio, Paul Lichtenstein, Ralf Kuja-Halkola, Isabell Brikell

**Affiliations:** 1 Department of Clinical Medicine, Aarhus University, Aarhus, Denmark; 2 Department of Affective Disorders, Aarhus University Hospital – Psychiatry, Aarhus, Denmark; 3 Department of Medical Epidemiology and Biostatistics, Karolinska Institutet, Stockholm, Sweden; 4 National Centre for Register-Based Research, Department of Public Health, Aarhus University, Aarhus, Denmark; 5 Bioinformatic Research Centre, Department of Molecular Biology and Genetics, Aarhus University, Aarhus, Denmark; 6 Novo Nordisk Foundation Center for Genomic Mechanisms of Disease, Broad Institute of MIT and Harvard, Cambridge, MA USA; 7 Department of Psychological and Brain Sciences, Indiana University, Bloomington, IN USA; 8 Department of Global Public Health and Primary Care, University of Bergen, Bergen, Norway; 9 Department of Biomedicine, Aarhus University, Aarhus, Denmark

**Keywords:** Psychiatric disorders, Genetics

## Abstract

Borderline personality disorder (BPD) often co-occurs with other health conditions, but the role of genetics vs. environment is unclear. In this multigenerational cohort study using Swedish national registers, the authors quantified the co-aggregation of BPD with other phenotypes in families and estimated the genetic vs. environmental contributions. In a birth cohort of individuals born 1973–2001 and their twins, siblings, cousins, parents, and aunts/uncles, the exposure was BPD in the proband. Odds ratios were estimated for 19 psychiatric, 18 somatic, and seven behavioral/injury outcome phenotypes within individuals and relative pairs. Structural equation modeling was used to estimate genetic, common environmental, and unique environmental contributions to phenotypic associations and genetic correlations (*r*_*g*_). Of the 2.7 million individuals in the birth cohort (mean age at follow-up end = 31.7 years, SD = 9.8, 48.5% female), 24,547 (86% female) were diagnosed with BPD by follow-up end. Relatives of individuals with BPD had increased risk for various psychiatric, somatic, and behavioral phenotypes, except cystic fibrosis. Psychiatric phenotypes showed the strongest phenotypic and genetic correlations, with equal contributions from genetic and unique environmental factors. The pattern varied for somatic phenotypes, which also had weaker correlations. Sleep disorders showed the strongest genetic overlap with BPD (*r*_*g*_ = 0.74, 95%CI = 0.39–1.08). Behavioral phenotypes related to BPD symptoms showed strong associations and genetic overlap (*e.g*., self-harm *r*_*g*_ = 0.80, 95%CI = 0.55–1.04), whereas accident-related phenotypes had weaker associations and varied genetic/environmental contributions. BPD co-aggregates with many phenotypes, with psychiatric conditions showing the strongest genetic overlap. These findings guide further research on BPD comorbidity causes and interventions.

## Introduction

Borderline personality disorder (BPD) is a psychiatric disorder characterized by pervasive instability of emotions, identity, and interpersonal relationships as well as impulsivity and self-harm [1]. Empirical studies on individuals with BPD have shown that adverse childhood experiences, especially sexual trauma, are common [2, 3]. However, genetics also play a role, with heritability estimates from family and twin studies ranging from 46–69% [4, 5]. In addition, BPD is known to have polygenic basis, with the to date largest genome-wide association study (GWAS) of BPD showing that common genetic variation explains a substantial proportion of heritability (single nucleotide polymorphism (SNP)-based heritability = 17%) [6].

The population prevalence of BPD is thought to be 0.7%–7.35% [7–9], with more female than male individuals receiving the diagnosis in clinical samples [10, 11], but a more even sex ratio observed in community-based survey studies [12, 13]. However, individuals with this diagnosis make up a large proportion of those in contact with the psychiatric healthcare system [14] and are also often treated for non-psychiatric conditions [15, 16]. High rates of comorbidity with many other psychiatric, somatic, and behavioral conditions, such as substance use disorders, cardiovascular disease, and accidents [17, 18], likely play a role in the high healthcare utilization and societal costs associated with BPD [19]. While some BPD comorbidity patterns have been shown to differ by sex, *e.g*., affective and anxiety disorders more commonly co-occurring in females than males and vice versa for substance disorders [20–22], the evidence for sex differences within other comorbidities is mixed and/or sparse. To date, it is not known why such high comorbidity is observed for BPD, and the associations likely arise from complex mechanisms.

One possible explanation for the observed associations between BPD and other phenotypes could be shared genetic factors. For example, BPD has been shown to have moderate to strong genetic correlations with post-traumatic stress disorder (PTSD), depression, attention-deficit hyperactivity disorder (ADHD), and chronic pain as well as weak genetic correlations with type 2 diabetes, body mass index, and asthma in a recent GWAS [23]. In addition, a national Swedish cohort study revealed that BPD and ADHD, which both share impulsivity and mentalization difficulties, co-occur within individuals and co-aggregate in families [24]. Further, the strength of the association between ADHD and BPD decreased along with decreasing genetic relatedness [24], suggesting an important role of genetic factors in this association. Such large-scale family studies can also be used to estimate genetic and environmental associations between phenotypes and are both a powerful alternative and complement to molecular genetic studies. This is because heritability, or the variance in a phenotype explained by genetic factors, is often lower when estimated with GWAS versus with family and twin studies, which reduces the power to detect co-heritability or genetic correlations between phenotypes [25, 26]. Despite their utility, studies using a genetically informative family design are largely lacking for most conditions associated with BPD.

In this study, we aimed to (1) provide an overview of the degree of within-individual co-occurrence and familial co-aggregation of BPD with a wide range of psychiatric, somatic, and behavioral phenotypes, and (2) use sibling data to estimate the contribution of genetic and environmental factors to the phenotypic associations in order to better understand the underlying mechanisms of BPD comorbidity.

## Methods and materials

### Study population

Data were obtained by linking multiple Swedish registries using the unique personal identification number assigned to all residents in Sweden. We created a birth cohort of all individuals born in Sweden between 1973-01-01 and 2001-12-31 with a known biological mother using the Swedish Medical Birth Register [27], excluding stillbirths and those born with congenital malformations linked to other health conditions (Supplementary Methods). Individuals were linked to their biological mothers, fathers, full siblings, maternal and paternal half-siblings, aunts, uncles, and first cousins using the Multi-Generation Register [28]. Information on twin zygosity was obtained from the Swedish Twin Registry [29]. Twins, siblings, and cousins were drawn from the same Swedish birth cohort as the probands, and thus each pair could be included twice in each familial co-aggregation analysis: *e.g*., first with BPD case/control status of relative A as the exposure and ADHD diagnosis of relative B as the outcome, then with BPD case/control status of relative B as the exposure and ADHD diagnosis of relative A as the outcome. We only included parents and aunts/uncles born 1933 or later to ensure adequate register coverage.

We obtained information on sex, immigrations, emigrations, and deaths from the Total Population Register [30] and the Cause of Death Register [31]. Individuals were followed from birth, first immigration to Sweden or the start of the International Classification of Diseases, Ninth Revision (ICD-9) in 1987-01-01 (whichever came last), until the first emigration, first registered diagnosis of the phenotype of interest, death, or 2020-12-31 (whichever came first). To allow enough time for probands to receive a diagnosis of the exposure (*i.e*., BPD) we excluded individuals who died or emigrated from Sweden prior to age 18.

This study was approved by the Swedish Ethical Review Authority (reference number 2020-06540). Informed consent is not required for pseudonymized register-based research according to the Swedish law.

### Borderline personality disorder

BPD was defined as ≥ 1 registered diagnosis of ICD-9 code 301.D or 301.J (1987–1996) or ICD-10-SE code F60.3 (since 1997) in the Swedish National Patient Register (NPR) [32, 33]. The NPR contains routinely collected data beginning in 1973 about discharge diagnoses and start/stop date of treatment from public inpatient and outpatient specialist care as a byproduct of the tax-funded universal healthcare system in Sweden (Supplementary Methods). The register-based BPD diagnosis has been shown to have a positive predictive value of 63%–100% depending on the use of ICD/Diagnostic and Statistical Manual of Mental Disorders criteria or expert opinion to determine true diagnosis from retrieved patient records [24, 34].

### Outcome phenotypes

We investigated the association between BPD and a total of 44 other phenotypes, including 19 psychiatric, 18 somatic, and seven behavioral/injury phenotypes defined using ≥ 1 registered primary or secondary diagnosis of their respective ICD codes in the NPR (Supplementary Tables S1–S3). Psychiatric phenotypes included alcohol use disorder, other substance use disorder, schizophrenia spectrum disorder, bipolar disorder, depressive disorder, anxiety disorder, obsessive-compulsive disorder, acute stress reaction, PTSD, adjustment disorder, anorexia nervosa, other eating disorder, other specific personality disorder, intellectual disability, autism spectrum disorder, ADHD, conduct disorder, childhood anxiety and emotional disorder, and tic disorder. Somatic phenotypes included epilepsy, cerebral palsy, migraine, sleep disorder, type 1 diabetes, type 2 diabetes, autoimmune disease, congenital hypothyroidism, cystic fibrosis, anaphylaxis, asthma, viral and/or bacterial infection, cardiovascular disease, polycystic ovary syndrome (PCOS), sexual pain, gastrointestinal problems, persistent somatic fatigue and body aches, and back, neck, and joint pain. Behavioral/injury phenotypes included accidental poisoning, transport-related accident, fall-related accident, traumatic brain injury, assault/victimization, self-harm, and death by suicide. Death by suicide was ascertained from the Cause of Death Register. Phenotypes were selected to include categories of conditions with prior evidence of association with BPD [18] as well as somatic conditions not previously studied.

### Statistical analysis

#### Within-individual and familial co-aggregation analysis

To describe the within-individual association of BPD with other phenotypes in the birth cohort, we used a generalized estimating equation with a logit link to estimate odds ratios (ORs), adjusting for sex and birth year.

To describe familial co-aggregation, we estimated ORs for each BPD-phenotype combination in different types of relative pairs, adjusting for sex, sex of relative, birth year, and birth year of the relative where applicable (Supplementary Methods). Due to changes in diagnostic practice over time and limited coverage for several childhood onset psychiatric disorders in the parental and aunt/uncle generation, we restricted analyses of psychiatric phenotypes to within-generation relative pairs (*i.e*., twins, full and half-siblings, and first cousins). For PCOS and sexual pain, henceforth referred to as female-only phenotypes, only female outcome individuals were included, and thus only sex of the relative acting as the exposure was adjusted for in the regression model where applicable.

To account for varying diagnostic trends and age at peak incidence for each phenotype, we adjusted for birth year using a natural cubic spline with five degrees of freedom using the *ns* function from the R package splines [35] (version 4.3.2). We calculated Wald-type 95% confidence intervals (95%CIs) using cluster-robust standard errors to account for the non-independence of family data (Supplementary Methods). We treated phenotypes as lifetime diagnoses, as previous studies have shown a considerable delay between symptom onset and official diagnosis for both psychiatric and somatic health conditions [36, 37].

#### Quantitative genetic analysis

We conducted structural equation modeling (SEM) with full sibling, maternal half-sibling, and paternal half-sibling pairs to gauge the contribution of shared additive genetic factors (A), common environmental factors (C) (*i.e*., factors that make members of the same family more similar), and unique environmental factors (E) to observed associations. Since the aim of the current study was to describe the patterns of these three factors (ACE) across multiple phenotypes, we did not fit reduced models (*e.g*., AE model), as different model specification between the phenotypes would bias estimates and impair comparison [38].

Quantitative genetic analysis was performed with sibling pairs to utilize large samples containing variation in degree of both shared genetics and family environment and to avoid between-generation differences in diagnostic trends. Full siblings share on average 50% of their DNA identical by descent, while maternal and paternal half-siblings share on average 25%. To estimate the common environmental contribution to the BPD-phenotype associations, we modeled C as being shared to an equal extent in full and maternal half-siblings (100%) and not shared by paternal half-siblings (0%). While this modeling assumption does not necessarily reflect reality across families, we can expect environmental factors that make relatives more similar to be reduced for siblings growing up in different households, which is the case for a larger extent of paternal half-siblings versus maternal half-siblings and full siblings [39]. One unique sibling pair of each type was selected from each independent family cluster (Supplementary Methods) using the following algorithm: (1) closest in age, (2) oldest, (3) random selection.

To guide further analytic decisions, we first performed univariate SEM to gauge the relative contribution of A, C, and E to each phenotype separately both with and without adjustment for sex and birth year (Supplementary Methods). Then, to quantify the degree to which the phenotypic correlations between BPD and other phenotypes could be explained by these factors, we conducted bivariate SEM (Supplementary Fig. S1) using the correlated factors approach and weighed least squares for model fitting [40, 41] to get the bivariate estimates (bivA, bivC, bivE). We adjusted for sex and birth year in the bivariate SEM, as the initial univariate SEM showed this adjustment to produce more conservative heritability estimates. For three phenotypes—anorexia nervosa, other specific personality disorders, and assault/victimization—we encountered optimization problems. Since the univariate SEM estimated C of approximately 0 for these phenotypes, we re-fitted the bivariate models with bivC fixed to 0 and did not estimate the common environmental correlation with BPD. In the bivariate models, but not in the univariate models, we constrained the univariate variance components to be positive.

We only performed bivariate SEM for phenotypes with some evidence of association with BPD (within-individual tetrachoric correlation ≥ 0.1) and, to facil with ≥ 5 discordant and concordant sibling pairs to facilitate modeling. Based on these criteria, congenital hypothyroidism, cystic fibrosis, cerebral palsy, type 1 diabetes, autoimmune disease, and back, neck, and joint pain were excluded. In total, 38 phenotypes were included in the quantitative genetic analyses.

#### Sensitivity analysis

To assess the potential impact of differential follow-up time, we also used Cox regression to estimat within-individual and between-relative associations as a sensitivity analysis, with BPD diagnosis treated as a time-varying exposure and attained age as the underlying time scale (Supplementary Methods).

Statistical analyses were performed between 2024-02-01 and 2024-10-15 at Karolinska Institutet in Stockholm, Sweden using R [35] (version 4.3.2) and packages drgee [42] (version 1.1.10), OpenMx [43] (version 2.21.11), and survival [44] (version 3.5.8).

## Results

The 2,665,478 individuals in the birth cohort had a mean age of 32.9 years at the end of follow-up (standard deviation = 8.3) after excluding those who died or emigrated from Sweden before the age of 18 years (18,829 and 101,376, respectively), and 24,073 (85.8% female) received a diagnosis of BPD, corresponding to a prevalence of 0.9% (Table 1, Supplementary Tables S4–S5). In total, 7088 unique monozygotic twin, 13,276 dizygotic twin, 1,434,189 full sibling, 2,632,129 mother-child, 2,602,867 father-child, 243,717 maternal half-sibling, 249,296 paternal half-sibling, 8,074,278 aunt/uncle-niece/nephew, and 5,433,963 first cousin pairs were identified for familial co-aggregation analysis. The reported results highlight patterns in point estimates, with Wald-type 95%CIs (which are not bound within the parameter space, such as −1 to 1 for correlations) included to qualify confidence in the estimates.

**Table 1:** Descriptive statistics for birth cohort.

	Total	Without BPD	With BPD
**N (percent)**	2,665,478	2,641,405 (99.1%)	24,073 (0.9%)
**Sex**			
Male	1,363,723	1,360,311 (99.7%)	3412 (0.3%)
Female	1,301,755	1,281,094 (98.4%)	20,661 (1.6%)
**Birth year**			
1973–1977	465,343	462,044 (99.3%)	3299 (0.7%)
1978–1982	424,509	420,446 (99.0%)	4063 (1.0%)
1983–1987	442,644	437,434 (98.8%)	5210 (1.2%)
1988–1992	544,529	538,404 (98.9%)	6125 (1.1%)
1993–1997	467,750	463,787 (99.2%)	3963 (0.8%)
1998–2001	320,703	319,290 (99.6%)	1413 (0.4%)
**Co-occurring phenotype**			
Alcohol use disorder	116,949	109,169 (4.1%)	7780 (32.3%)
Drug use disorder	86,275	77,165 (2.9%)	9110 (37.8%)
Schiz. spectrum disorder	25,931	23,149 (0.9%)	2782 (11.6%)
Bipolar disorder	38,789	32,398 (1.2%)	6391 (26.5%)
Depressive disorder	237,283	218,851 (8.3%)	18,432 (76.6%)
Anxiety disorder	109,073	99,742 (3.8%)	9331 (38.8%)
OCD	32,427	29,663 (1.1%)	2764 (11.5%)
Acute stress reaction	63,903	57,053 (2.2%)	6850 (28.5%)
PTSD	26,522	21,602 (0.8%)	4920 (20.4%)
Adjustment disorder	76,281	69,895 (2.6%)	6386 (26.5%)
Anorexia nervosa	15,709	14,091 (0.5%)	1618 (6.7%)
Other eating disorder	33,211	28,827 (1.1%)	4384 (18.2%)
Other specific PD	10,296	7738 (0.3%)	2558 (10.6%)
Intellectual disability	186,122	175,695 (6.7%)	10,427 (43.3%)
Autism spectrum disorder	56,043	53,255 (2.0%)	2788 (11.6%)
ADHD	137,632	128,488 (4.9%)	9144 (38.0%)
Conduct disorder	13,501	12,240 (0.5%)	1261 (5.2%)
Childhood anxiety	3156	2855 (0.1%)	301 (1.3%)
Tic disorder	8372	8156 (0.3%)	216 (0.9%)
Epilepsy	40,125	39,067 (1.5%)	1058 (4.4%)
Cerebral palsy	6,902	6844 (0.3%)	58 (0.2%)
Migraine	81,330	79,515 (3.0%)	1815 (7.5%)
Sleep disorder	87,005	82,079 (3.1%)	4926 (20.5%)
Type 1 diabetes	26,997	26,559 (1.0%)	438 (1.8%)
Type 2 diabetes	13,744	13,286 (0.5%)	458 (1.9%)
Autoimmune disease	163,316	160,937 (6.1%)	2379 (9.9%)
Congen. hypothyroid.	1649	1614 (0.1%)	35 (0.1%)
Cystic fibrosis	581	576 (0.0%)	5 (0.0%)
Anaphylaxis	18,362	17,995 (0.7%)	367 (1.5%)
Asthma	211,592	208,236 (7.9%)	3356 (13.9%)
Infections	1,024,938	1,009,137 (38.2%)	15,801 (65.6%)
CVD	116,065	113,957 (4.3%)	2108 (8.8%)
PCOS^a^	30,063	29,159 (2.3%)	904 (4.4%)
Sexual pain^a^	138,182	133,421 (10.4%)	4761 (23.0%)
Gastroint. problems	75,639	73,690 (2.8%)	1949 (8.1%)
Fatigue / aches	10,960	10,223 (0.4%)	737 (3.1%)
Joint pain	374,609	369,684 (14.0%)	4925 (20.5%)
Accidental fall	909,460	898,744 (34.0%)	10,716 (44.5%)
Accidental poisoning	52,102	49,703 (1.9%)	2399 (10.0%)
Transport-related accident	384,441	379,131 (14.4%)	5310 (22.1%)
Traumatic brain injury	265,369	261,022 (9.9%)	4347 (18.1%)
Assault / victimization	102,167	98,054 (3.7%)	4113 (17.1%)
Self-harm	114,112	102,349 (3.9%)	11,763 (48.9%)
Suicide	6933	6297 (0.2%)	636 (2.6%)

Percentages correspond to the row-wise sums except for co-occurring phenotypes, where the percentages correspond to the sum per with/without BPD group.

*OCD* obsessive compulsive disorder, *PTSD* post- traumatic stress disorder, *PD* personality disorder, *ADHD* attention-deficit hyperactivity disorder, *CVD* cardiovascular disease, *PCOS* polycystic ovary syndrome.

^a^Only ascertained for individuals with female sex.

**Fig. 1:**
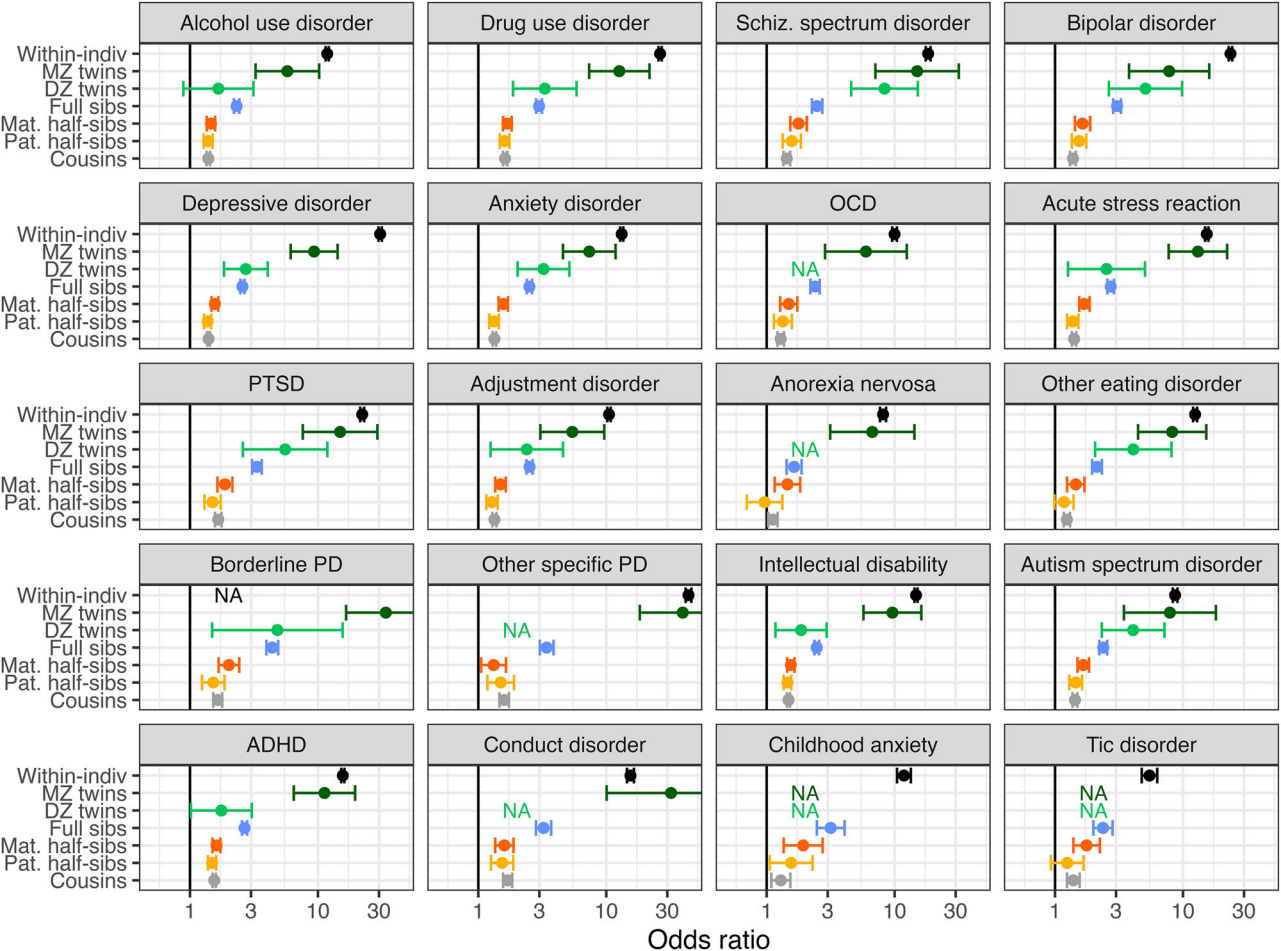
Odds ratio of psychiatric phenotype diagnosis when having a borderline personality disorder diagnosis oneself, or a relative diagnosed with borderline personality disorder. Odds ratios are adjusted for sex, sex of relative, birth year, and birth year of relative where applicable and shown with 95% Wald-type confidence intervals based on cluster-robust standard errors. X-axis is log-transformed and scaled to fit the minimum and maximum point estimates observed for all phenotypes studied. NA not available (indicates that there were too few observations to estimate the association), Within-indiv within-individual association, MZ *twins* monozygotic twins, DZ *twins* dizygotic twins, Mat. maternal, Pat. paternal, Sibs siblings, OCD obsessive compulsive disorder, PTSD post-traumatic stress disorder, PD personality disorder, ADHD attention-deficit hyperactivity disorder.

**Fig. 2:**
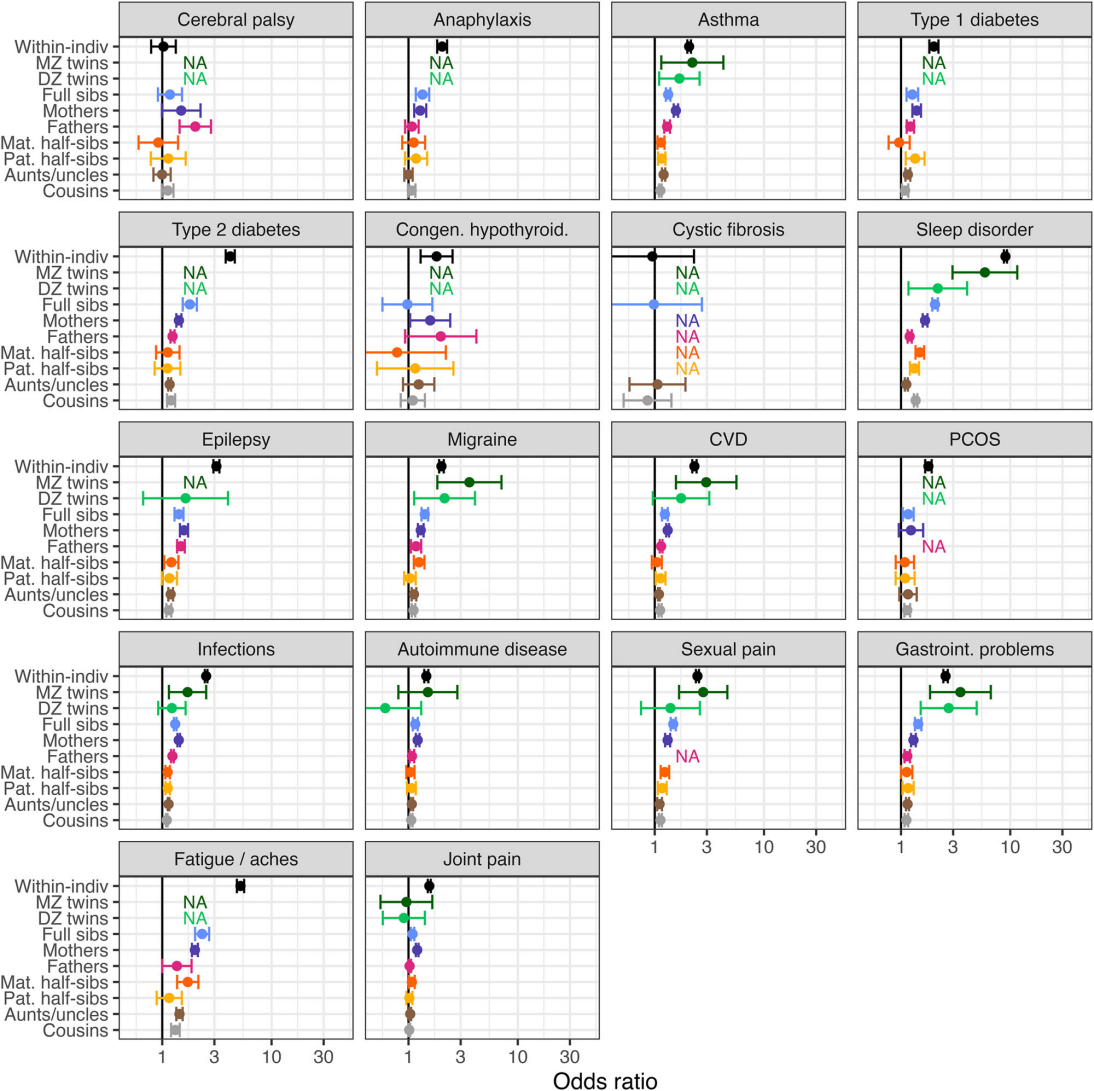
Odds ratio of a somatic phenotype diagnosis when having a borderline personality disorder diagnosis oneself, or a relative diagnosed with borderline personality disorder. Odds ratios are adjusted for sex, sex of relative, birth year, and birth year of relative where applicable and shown with 95% Wald-type confidence intervals based on cluster-robust standard errors. X-axis is log-transformed and scaled to fit the minimum and maximum point estimates observed for all phenotypes studied. NA not available (indicates that there were too few observations to estimate the association), Within-indiv within-individual association, MZ *twins* monozygotic twins, DZ *twins* dizygotic twins, Mat. maternal, Pat. paternal, Sibs siblings, CVD cardiovascular disease, PCOS polycystic ovary syndrome.

**Fig. 3:**
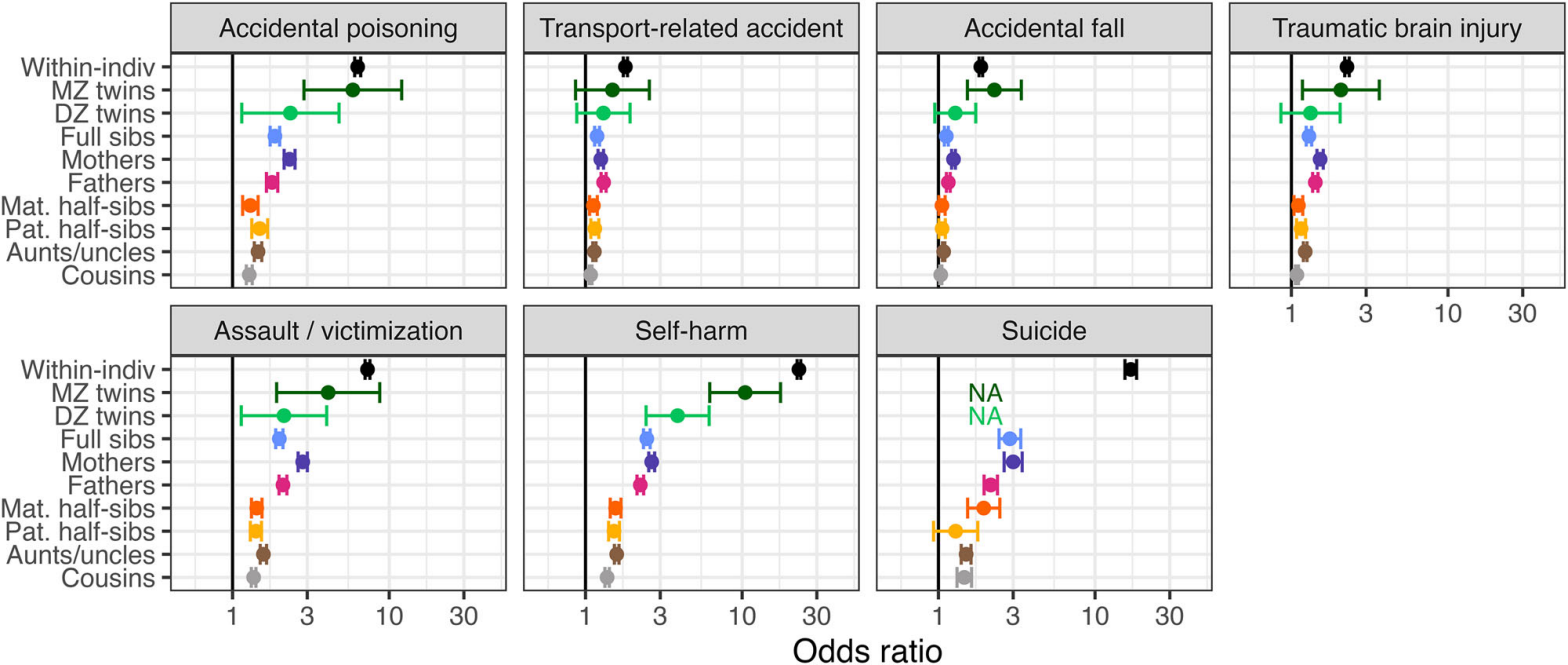
Odds ratio of a behavioral/injury phenotype diagnosis when having a borderline personality disorder diagnosis oneself, or a relative diagnosed with borderline personality disorder. Odds ratios are adjusted for sex, sex of relative, birth year, and birth year of relative where applicable and shown with 95% Wald-type confidence intervals based on cluster-robust standard errors. X-axis is log-transformed and scaled to fit the minimum and maximum point estimates observed for all phenotypes studied. NA not available (indicates that there were too few observations to estimate the association), Within-indiv within-individual association, MZ *twins* monozygotic twins, DZ *twins* dizygotic twins, Mat. maternal, Pat*.* paternal, Sibs siblings.

Within individuals, BPD was associated with all psychiatric phenotypes (OR range = 5.5–43.9), all somatic phenotypes except for cystic fibrosis and cerebral palsy (OR range = 1.0–9.0), and all behavioral/injury phenotypes (OR range = 1.8–23.1) (Figs. 1–3). The strongest within-individual associations were observed for other specific personality disorder and depressive disorder in the psychiatric domain, sleep disorder and chronic body aches/fatigue in the somatic domain, and self-harm and suicide in the behavioral domain.

### Familial co-aggregation analysis

We observed a clear pattern of familial co-aggregation between BPD and all **psychiatric phenotypes**, with associations generally decreasing with decreasing genetic relatedness, many strong associations, and few null associations (Fig. 1, Supplementary Table S6). The psychiatric phenotype with the most pronounced association with BPD across relative pairs was PTSD. A weak-to-strong association was observed across all relative pairs, decreasing with decreasing relatedness (monozygotic twin: OR = 14.9, 95%CI = 7.6–29.2; dizygotic twin: OR = 5.5, 95%CI = 2.6–11.8; full sibling: OR = 3.3, 95%CI = 3.1–3.6; maternal half-sibling: OR = 1.9, 95%CI = 1.6–2.1; paternal half-sibling: OR = 1.5, 95%CI = 1.3–1.7; cousin: OR = 1.7, 95%CI = 1.6–1.8). After PTSD, the psychiatric phenotypes with at least a weak association with BPD across all relative pairs were drug use disorder, intellectual disability, and conduct disorder. However, for specific relative pairs, the strongest associations were with other disorders: for example, for full siblings, the strongest association was with other specific personality disorder (OR = 3.4, 95%CI = 3.0–3.9). The few psychiatric phenotypes showing null associations and associations not even reaching weak strength in select relative pairs despite evidence of strong within-individual associations consisted of tic disorder, other eating disorder, and anorexia nervosa.

Evidence of familial co-aggregation with BPD was more varied for **somatic phenotypes**, and the associations were generally weaker than for psychiatric phenotypes (Fig. 2, Supplementary Table S7). The somatic phenotype with the most pronounced association with BPD across relative pairs was sleep disorders. A strong association among monozygotic twins (OR = 5.9, 95%CI = 3.0–11.6) and weak association among dizygotic twin (OR = 2.2, 95%CI = 1.2–4.0), full sibling (OR = 2.0, 95%CI = 1.0–2.2), mother-child (OR = 1.7, 95%CI = 1.6–1.7), and maternal half-sibling pairs (OR = 1.5, 95%CI = 1.4–1.6) was observed between BPD and sleep disorders, with the remaining relative pair estimates consistently non-null. Moderate associations with BPD were observed in monozygotic twin pairs for migraine, gastrointestinal problems, cardiovascular disease, and sexual pain, with the associations decreasing along with decreasing genetic relatedness but not consistently non-null across other types of relative pairs for these phenotypes. Weak, but consistently non-null associations across relative pairs were observed for asthma. Some weak associations with BPD were observed for type 2 diabetes, epilepsy, infection, and chronic body aches/fatigue, with the associations decreasing along with decreasing genetic relatedness but not consistently non-null across relative pairs. Anaphylaxis, type 1 diabetes, PCOS, autoimmune disease, and back, neck, and joint pain did not show a pattern of familial co-aggregation with BPD despite some non-null associations observed in select relative pairs. Congenital hypothyroidism, which was weakly associated with BPD within individuals, showed only null associations with BPD across relative pairs. We found evidence of neither within-individual association nor familial co-aggregation between BPD and cystic fibrosis or cerebral palsy, except a weak association with cerebral palsy in father-child pairs (OR = 2.0, 95%CI = 1.4–2.8).

Among the **behavioral/injury phenotypes**, we observed a clear pattern of familial co-aggregation of BPD with accidental poisoning, assault/victimization, and self-harm, whereas associations were weaker and less consistent for transport-related accident, accidental fall, and traumatic brain injury (Fig. 3, Supplementary Table S8). Death by suicide showed weak-to-moderate but also one null association with BPD across relative pairs. The most pronounced associations were observed for self-harm, where weak-to-strong associations with BPD were observed across all relative pairs but cousins (monozygotic twin: OR = 10.5, 95%CI = 6.2–17.6; dizygotic twin: OR = 3.9, 95%CI = 2.4–6.2; full sibling: OR = 2.5, 95%CI = 2.3–2.6; mother-child: OR = 2.6, 95%CI = 2.5–2.6; father-child: OR = 2.2, 95%CI = 2.1–2.4; maternal half-sibling: OR = 1.6, 95%CI = 1.4–1.7; paternal half-sibling: OR = 1.5, 95%CI = 1.4–1.6; aunt/uncle-niece/nephew: OR = 1.6, 95%CI = 1.5–1.6; cousins: OR = 1.4, 95%CI = 1.3–1.4).

### Quantitative genetic analysis

We retained 765,171 unique full sibling, 91,475 maternal half-sibling, and 85,399 paternal half-sibling pairs from independent family clusters for quantitative genetic analysis. In the univariate analyses, the 95%CI of the phenotypic variance explained by common environmental factors included zero for 24 out of the 38 phenotypes, including BPD (Supplementary Table S9).

**Fig. 4:**
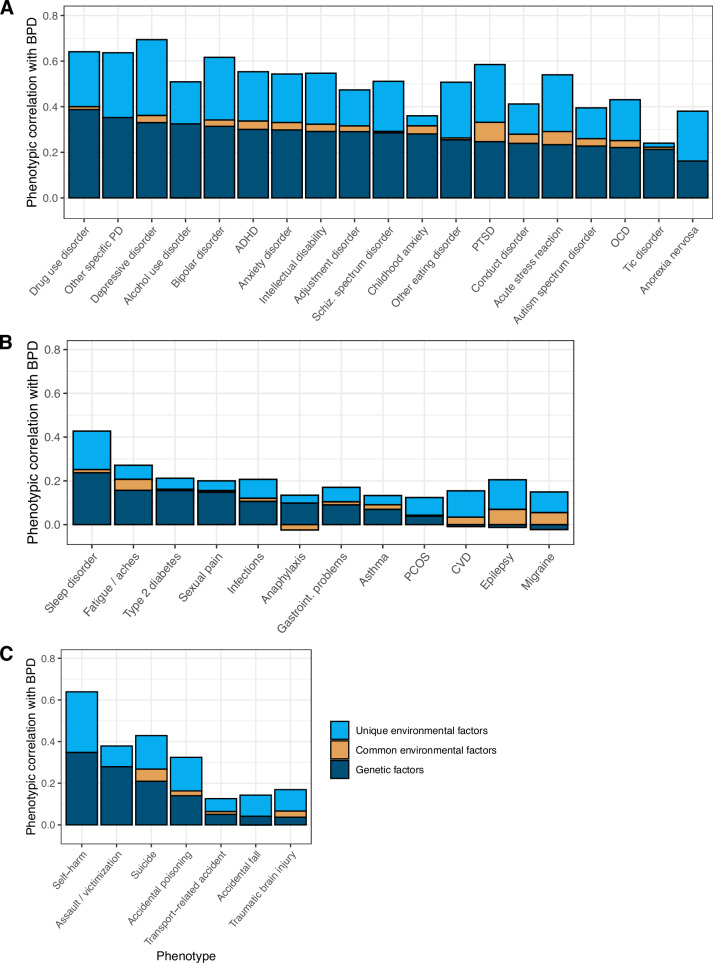
Contributions of genetic, common environmental, and unique environmental factors to the phenotypic correlations between borderline personality disorder and other phenotypes. The proportion of the phenotypic correlation with borderline personality disorder (BPD) explained by shared additive genetic factors, common environmental factors, and unique environmental factors for domains psychiatric **A**, somatic **B**, and behavioral/injury **C** phenotypes. The y-axis is the phenotypic correlation with BPD. Estimates are based on 765,171 unique full sibling, 91,475 maternal half-sibling, and 85,399 paternal half-sibling pairs from independent family clusters and ordered by absolute contribution of additive genetic factors. Negative estimates reflect etiological factors that have negative contributions to the proportion of phenotypic correlation. Structural equation modeling was not performed for congenital hypothyroidism, cystic fibrosis, cerebral palsy, type 1 diabetes, autoimmune disease, and back, neck, and joint pain, as these phenotypes had a within-individual phenotypic correlation with BPD < 0.1 and/or too few observations of discordant and concordant sibling pairs. Female-only analyses (PCOS and sexual pain) were conducted with a reduced dataset of 244,000 full sibling, 35,512 maternal half-sibling, and 35,257 paternal half-sibling female same-sex pairs. PD personality disorder, PTSD post-traumatic stress disorder, ADHD attention deficit hyperactivity disorder, OCD obsessive-compulsive disorder, PCOS polycystic ovary syndrome, CVD cardiovascular disease.

In the bivariate analyses, we observed that the phenotypic correlations between BPD and other phenotypes were, on average, explained half by genetic factors (bivA) and half by unique environmental (bivE) factors, with a minimal role of common environmental factors (bivC) (Fig. 4, Supplementary Table S10).

Across **psychiatric phenotypes**, genetic factors explained 42%–89% of the BPD-phenotype associations, common environmental factors 0%–14%, and unique environmental factors 7%–57% (Fig. 4A). The proportion of BPD-phenotype association explained by genetic factors was greatest for tic disorder and childhood anxiety and emotional disorder, albeit with very large confidence intervals: bivA = 89%, 95%CI = −8%–185% and bivA = 78%, 95%CI = −8%–164%, respectively. Compared to other psychiatric phenotypes, PTSD showed the greatest proportion of the phenotypic association with BPD explained by common environmental factors (bivC = 14%, 95%CI = 4%–25%). The phenotype with the greatest proportion of the association with BPD explained by unique environmental factors was anorexia nervosa (bivE = 57%, 95%CI = 34%–81%).

Across **somatic phenotypes**, genetic factors explained −18% to 90% of the BPD-phenotype associations, while common environmental factors explained −22% to 44% and unique environmental factors 22%–83% (Fig. 4B). The proportion of BPD-phenotype association explained by genetic factors was greatest for anaphylaxis (bivA = 90%, 95%CI = −92%–272%) and sexual pain (bivA = 74%, 95%CI = 28%–120%), again with very large 95%CIs. Compared to other somatic phenotypes, migraine showed the greatest proportion explained by common environmental factors (bivC = 44%, 95%CI = 3%–84%). The association between BPD and cardiovascular disease had the greatest proportion explained by unique environmental factors (bivE = 83%, 95%CI = 36%–129%).

Across **behavioral phenotypes**, genetic factors explained 22%–74%, common environmental factors -1% to 18%, and unique environmental factors 26%–71% of the BPD-phenotype associations (Fig. 4C). The greatest proportion of the phenotypic association with BPD explained by genetic factors was observed for assault/victimization (bivA = 74%, 95%CI = 63%–85%). Traumatic brain injury had the greatest proportion of association with BPD explained by common environmental factors (bivC = 18%, 95%CI = -3%–38%), while accidental falls had the greatest proportion explained by unique environmental factors (bivE = 71%, 95%CI = 44%–99%).

**Fig. 5:**
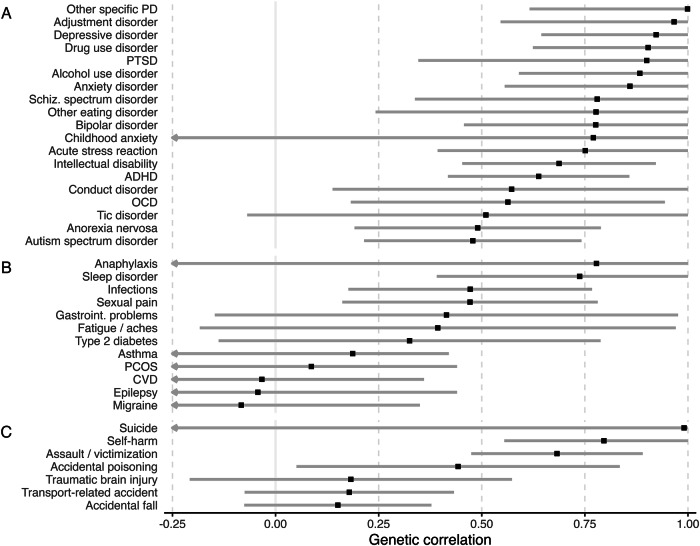
Genetic correlations of borderline personality disorder with other phenotypes. Genetic correlations and 95% confidence intervals derived from bivariate structural equation modeling between borderline personality disorder (BPD) and other psychiatric **A**, somatic **B**, and behavioral/injury **C** phenotypes. Estimates are based on 765,171 unique full sibling, 91,475 maternal half-sibling, and 85,399 paternal half-sibling pairs from independent family clusters and ranked by the strength of the genetic correlation. Structural equation modeling was not performed for congenital hypothyroidism, cystic fibrosis, cerebral palsy, type 1 diabetes, autoimmune disease, or back, neck, and joint pain, as these phenotypes had a within-individual phenotypic correlation with BPD < 0.1 and/or too few observations of discordant and concordant sibling pairs. Female-only analyses (PCOS and sexual pain) were conducted with a reduced dataset of 244,000 full sibling, 35,512 maternal half-sibling, and 35,257 paternal half-sibling female same-sex pairs. PD personality disorder, PTSD post-traumatic stress disorder, ADHD attention deficit hyperactivity disorder, OCD obsessive-compulsive disorder, PCOS polycystic ovary syndrome, CVD cardiovascular disease.

Genetic correlations derived from the bivariate quantitative genetic analyses suggested considerable overlap in genetic etiology between BPD and most psychiatric phenotypes (*r*_g_ = 0.48–1.00) and genetic overlap between BPD and some of the somatic (*r*_g_ = −0.08–0.78) and behavioral (*r*_g_ = 0.15–0.99) phenotypes (Fig. 5, Supplementary Table S10).

Across **psychiatric phenotypes**, the strongest genetic correlations with BPD were observed for other specific personality disorder (*r*_g_ = 1.00, 95%CI = 0.62–1.38), adjustment disorder (*r*_g_ = 0.97, 95%CI = 0.55–1.39), and depressive disorder (*r*_g_ = 0.92, 95%CI = 0.64–1.20) (Fig. 5A), and most other phenotypes showed moderate-to-strong correlations. The weakest genetic correlations with BPD were observed for autism spectrum disorder (*r*_g_ = 0.48, 95%CI = 0.21–0.74) and anorexia nervosa (*r*_g_ = 0.49, 95%CI = 0.19–0.79), while null correlations were observed for childhood anxiety and emotional disorder (*r*_g_ = 0.77, 95%CI = −0.25–1.79) and tic disorder (*r*_g_ = 0.51, 95%CI = −0.07–1.09).

Across **somatic phenotypes**, a strong genetic correlation with BPD was observed for sleep disorder (*r*_g_ = 0.74, 95%CI = 0.39–1.08) (Fig. 5B). Infection (*r*_g_ = 0.47, 95%CI = 0.18–0.77) and sexual pain (*r*_g_ = 0.47, 95%CI = 0.16–0.78) also showed moderate genetic correlations with BPD. All other genetic correlation estimates for somatic phenotypes included null in the confidence interval.

Across **behavioral phenotypes**, strong genetic correlations with BPD were observed for self-harm (*r*_g_ = 0.80, 95%CI = 0.55–1.04) and assault/victimization (*r*_g_ = 0.68, 95%CI = 0.47–0.89) (Fig. 5C). Accidental poisoning showed a moderate genetic correlation with BPD (*r*_g_ = 0.44, 95%CI = 0.05–0.83). All other genetic correlation estimates for behavioral/injury phenotypes included null in the confidence interval.

### Sensitivity analysis

Estimates from the sensitivity analysis with BPD defined as a time-varying exposure closely followed the pattern of results from the logistic regression analysis (Supplementary Tables S6–S8, Supplementary Figures S2–S4). In general, we observed wider confidence intervals around the hazard ratios and an increased number of associations that could not be estimated due to the exclusion of prevalent cases (*e.g*., exclusion of relative pair due to relative B receiving a cystic fibrosis diagnosis before relative A reached start of follow-up / 18 years of age) in the sensitivity analysis. The association between BPD and cerebral palsy in father-child pairs was no longer found in the sensitivity analysis (HR = 1.5, 95%CI = 0.7–3.2). Within-individual associations of BPD with mood, substance abuse, anxiety, stress, and other eating disorder as well as ADHD, infection, and self-harm decreased while those with autism, epilepsy, asthma, accidental poisoning, traumatic brain injury, and suicide as well as conduct and tic disorder increased in the sensitivity analysis.

## Discussion

To our knowledge, this is the first large-scale study to describe patterns of familial co-aggregation and use bivariate quantitative genetics to understand the association of BPD with a wide range of phenotypes. We found familial co-aggregation of BPD with many phenotypes and non-null associations even between first cousins. Psychiatric conditions showed the strongest associations and most consistent genetic overlap. Associations with somatic and behavioral phenotypes were weaker, with greater variation in genetic and environmental contributions. These findings provide a roadmap for further investigating the underlying mechanisms of, and potential interventions for, comorbidity in BPD.

Our results suggest that unique environmental and genetic factors are equally important for the familial co-aggregation of BPD with other psychiatric phenotypes, whereas the common environment plays a minimal role. Despite this, familial co-aggregation estimates were often slightly stronger for maternal half-siblings than paternal half-siblings, suggesting some effect of the common environment. Common environmental factors contributed the most to the association between BPD and PTSD, which may be expected: exposure to a stressful event of catastrophic nature (such as war or physical or sexual abuse), a diagnostic criterium for PTSD and frequently reported experience among individuals with BPD [2, 3], is more likely to be shared by members of the same family. However, the strong genetic correlation (*r*_g_ = 0.90) suggests that familial genetic risk is also important. For the association between BPD and ADHD, the estimated contribution of additive genetic (54%) and unique environmental factors (39%) from our study is comparable with the results of a study in both twin and non-twin sibling pairs using borderline personality traits and ADHD symptoms (49% and 51%, respectively) [45]. However, whereas we also estimated the contribution of common environmental factors (C) to this association (7%), the previous study used an ADE model, which ignores C and instead includes dominant genetic effects.

Genetic factors seem to play a large role in the association between BPD and depression, and the strong genetic correlation suggests a considerable overlap in the underlying genetic mechanisms. The strong genetic correlations with several psychiatric phenotypes in this study are consistent with those reported in the recent BPD GWAS where phenotype definitions match and a sufficiently large discovery sample size is available [23]. The large role of shared genetics in the association between BPD and many other psychiatric disorders in this study is in line with molecular genetic studies showing that psychiatric disorders are complex heritable traits sharing underlying genetic architecture, possibly due to both pleiotropy and cross-trait assortative mating [46, 47]. However, the observed associations may also reflect classification difficulties, as many psychiatric disorders have overlapping symptoms. This may especially be the case for other specific personality disorders and PTSD, which share symptoms with BPD. Future studies investigating the association between BPD and other psychiatric disorders would benefit from symptom-level analysis to disentangle shared etiology of distinct symptoms from symptom overlap of categorical diagnoses. Furthermore, careful interpretation of the quantitative genetic results is needed given the large degree of statistical uncertainty in the estimates.

The findings for somatic phenotypes were more varied and represent novel avenues for investigation. A clear null finding was cystic fibrosis, a rare recessive genetic disorder, as all associations with BPD were approximately null. While not associated with BPD within individuals, cerebral palsy risk was found to be higher in the fathers of probands with BPD (although not in the sensitivity analysis), a finding difficult to interpret given the heterogeneous etiology of the diagnosis and lack of research about the children of individuals with cerebral palsy [48, 49]. Despite evidence of within-individual association with BPD, no consistent pattern of familial co-aggregation was found for type 1 diabetes, autoimmune disease, congenital hypothyroidism, anaphylaxis, PCOS, or back, neck, and joint pain. Of note, the negative bivariate variance component point estimates for anaphylaxis as well as epilepsy, migraine, and cardiovascular disease are likely induced by the rare combination of these phenotypes with BPD in sibling pairs. Future studies investigating potential causal links between BPD and these conditions might therefore benefit from examining mechanisms directly related to living with BPD and/or whether individuals with BPD simply are more likely to be in contact with healthcare services (and thus have other health conditions diagnosed) than those without.

However, in agreement with previous research showing increased co-occurrence of BPD with a range of somatic conditions [17, 18], the remaining 10 somatic phenotypes included in this study showed some evidence of familial co-aggregation with BPD, with a varied pattern of genetic and environmental contributions to the associations. Due to the large degree of statistical uncertainty in the bivariate SEM estimates for the somatic disorders especially, these should be interpreted with caution. Sleep disorder, where the strong phenotypic association with BPD could be attributed to an approximately equal contribution of genetic and unique environmental factors (and minimal contribution of common environment factors), also had the strongest genetic correlation with BPD (*r*_g_ = 0.74). These findings indicate that the underlying genetic effects of sleep disorders [50] and BPD may overlap and thus contribute to the well-documented but complex association between sleep disturbances and BPD symptoms [51, 52]. A similar picture also emerged for viral and/or bacterial infection, albeit with a less consistent pattern of weaker phenotypic correlations and genetic correlation. Inflammatory dysregulation, which has been described in individuals with BPD and hypothesized to be a potential contributory mechanism in various forms of psychopathology via stress [53], may increase the risk of infection [54]. In contrast to the recent BPD GWAS, which reported a weak genetic correlation with migraine [23], we found observed a null genetic correlation, possibly due to the rarity of migraine cases treated in specialist care in Sweden. Our findings also suggest that primarily environmental factors (both those shared within families and unique to individuals) contribute to the co-occurrence of BPD and cardiovascular disease, with little to no genetic contribution, suggesting that the observed association of cardiovascular disease with BPD could be amenable to environmental intervention. Future studies using molecular genetic approaches could be used to explore the etiological overlap of BPD and somatic conditions where genetic factors play a larger role, as molecular genetic findings from large samples are already available for many somatic conditions [55, 56].

The behavioral/injury phenotypes all showed some evidence of familial co-aggregation with BPD. Phenotypes constituting symptoms (*i.e*., self-harm, suicide) [1] and known risk factors (*i.e*., assault/victimization) [2, 3] of BPD generally showed the strongest associations and considerable genetic overlap (albeit with a large confidence interval for suicide due to its rarity), whereas accident-related phenotypes showed weaker to null phenotypic associations and more varied contribution of genetic and environmental factors. In line with estimates of genetic and unique environmental correlations between childhood trauma and BPD traits (0.53 and 0.11, respectively) from a previous twin study using a bivariate AE model [57], we found assault/victimization, which includes physical/sexual/emotional abuse in both childhood and adulthood, to have similar genetic and unique environmental correlations with BPD (*r*_g_ = 0.68; *r*_e_ = 0.18), with the contribution of shared environment fixed to zero. Accidental poisoning, which showed the highest genetic correlation with BPD among accident-related phenotypes, may be capturing some misclassified self-harm/suicide attempts [58] and harmful substance use, both associated with BPD [59, 60]. Within-individual estimates for death by suicide and self-harm were markedly stronger than familial co-aggregation estimates, suggesting that unique environmental or individual-specific factors rather than familial risk may be important to consider when attempting to prevent these outcomes. Familial co-aggregation estimates were slightly higher for dizygotic twins than full siblings across a wide range of not only behavioral, but also psychiatric and somatic phenotypes, suggesting a possible twin effect due to, *e.g*., sharing environments more similarly than regular siblings and different prenatal environments in twin versus non-twin pregnancies [61].

The strengths of this study include the lack of recall bias, clinical diagnoses, extensive follow-up time, and large sample size afforded by using Swedish national register data. While community-based samples from Sweden have estimated a higher prevalence of BPD (~ 5%) using questionnaires [62], the prevalence of BPD in the birth cohort in this study (0.9%) falls in the lower, albeit normal range from meta-analyses, and is to be expected given the inclusion of individuals treated in healthcare settings. Furthermore, while past studies investigating BPD etiology have focused on twins [5], which are relatively uncommon, this study uses many different types of biological relatives and a large sibling sample for quantitative genetic analysis.

The results of this study should be interpreted in the context of the following limitations. First, we likely capture individuals with more severe phenotypes, as the NPR only includes diagnoses from specialist care. These individuals may have a higher burden of comorbidity, thus leading to higher associations between phenotypes. At the same time, we may underestimate within-individual associations with conditions such as depression and infection, where a proportion of cases are only ever treated in primary care and thus not registered in the NPR. Potential biases in between-relative associations are harder to deduce, and these could potentially differ for co-aggregation among different relatives. Second, the findings may not generalize to other contexts, as culture, healthcare system, and diagnostic practice can influence the measured prevalence of phenotypes. Third, the birth cohort was selected to study BPD and may not be ideally suited to investigate all phenotypes for all relative pairs included in this study. For example, the oldest individuals in the birth cohort were 48 years old at end of follow-up, an age at which cardiovascular disease is still relatively rare, meaning our bivariate quantitative genetic results are limited to early-onset cardiovascular disease and may not generalize. Additionally, inferences should consider that we conditioned on being alive and living in Sweden until age 18 in the main analysis to allow enough time to be diagnosed with BPD and other phenotypes, suggesting that findings may not extend to some early-life outcomes. Fourth, follow-up time differed between individuals, which can also introduce bias. However, accounting for this in the sensitivity analysis did not change the overall conclusions. Fifth, since the unique environmental component in quantitative genetic analysis captures residual variance not explained by genetic or common environmental effects as well as different types of measurement error, correlated measurement error between phenotypes (*e.g*., correlated probability of diagnosis due to general health seeking behavior) could lead to upward biased within-individual estimates. Sixth, while it is likely that our estimates of genetic overlap between BPD and some psychiatric phenotypes from full and half-sibling pairs are inflated due to assortative mating [47], it is less clear if this also plays a role for somatic and behavioral phenotypes and likely varies by phenotype. Finally, this study identifies associations suggestive of mechanisms of shared and non-shared etiology but does not allow any conclusions to be made about the causal direction of these associations. While beyond the scope of this study, future studies could use more complex family designs and causal epidemiological methods to investigate and try to account for some of the abovementioned limitations [63, 64].

In conclusion, within individuals and between relatives, BPD was most strongly associated with other psychiatric phenotypes, and these associations were quite consistently explained in equal part by genetic and unique environmental factors, with little to no role of common environmental factors. The findings for somatic phenotypes were weaker and far more varied in terms of contribution of genetic and environmental factors, with several phenotypes showing little to no contribution of genetic factors. Behavioral phenotypes closely linked to BPD diagnostic criteria and risk factors showed stronger familial co-aggregation and evidence for shared genetic underpinnings, whereas phenotypes related to accidents and injuries showed weaker associations and a more varied pattern of genetic and environmental contributions. These findings are important for improving our understanding of the underlying mechanisms of BPD comorbidity.

## Supplementary information

Supplementary Information

## Data Availability

The data analyzed in this study are available from Statistics Sweden and The Swedish National Board of Health and Welfare. The Public Access to Information and Secrecy Act in Sweden prohibits publication of individual-level data. Researchers who are interested in replicating our work can, after obtaining ethical approval, apply for data through Statistics Sweden at: https://www.scb.se/en/services/guidance-for-researchers-and-universities/.
