# Supplementary material for: Borderline personality disorder and other psychiatric, somatic, and behavioral conditions: a nationwide family study
Source: Transl Psychiatry. 2026 Apr 10;16:257. doi: 10.1038/s41398-026-04001-w (PMC13184003; doi:10.1038/s41398-026-04001-w)
Supplement: Supplementary file 1 — Supplementary Information [file 41398_2026_4001_MOESM1_ESM.pdf]

Supplementary Information

Hall *et al*. Borderline personality disorder and psychiatric, somatic, and behavioral outcomes: A nationwide family study.

Supplementary Methods

Figure S1. Bivariate structural equation model using different sibling pairs (correlated factors approach)

Table S1. ICD codes used to ascertain psychiatric phenotypes

Table S2. ICD codes used to ascertain somatic phenotypes

Table S3. ICD codes used to ascertain behavioral phenotypes and injuries

Table S4. Number of registered diagnoses for borderline personality disorder cases in birth cohort

Table S5. Birth year and age at end of follow-up of different cohorts

Table S6. Familial co-aggregation results for psychiatric phenotypes with borderline personality disorder

Table S7. Familial co-aggregation results for somatic phenotypes with borderline personality disorder

Table S8. Familial co-aggregation results for behavioral / injury phenotypes with borderline personality disorder

Table S9. Univariate structural equation modeling results of quantitative genetic analysis

Table S10. Bivariate structural equation modeling results of quantitative genetic analysis with borderline personality disorder

Figure S2. Hazard ratio of psychiatric phenotype diagnosis when having a borderline personality disorder diagnosis oneself, or a relative diagnosed with borderline personality disorder

Figure S3. Hazard ratio of somatic phenotype diagnosis when having a borderline personality disorder diagnosis oneself, or a relative diagnosed with borderline personality disorder

Figure S4. Hazard ratio of behavioral/injry phenotype diagnosis when having a borderline personality disorder diagnosis oneself, or a relative diagnosed with borderline personality disorder

Supplementary References

Supplementary Methods

Study population

The birth cohort of 2,785,683 individuals contains all everyone born in Sweden between 1973-01-01 and 2001-12-31 with a known biological mother, excluding stillbirths (12,036) and those with congenital malformations (125,717). Stillborn children are included in the Swedish Medical Birth Register (1) for pregnancies of ≥28 weeks in length up until 2008-06-30 and for pregnancies of ≥22 weeks in length from 2008-07-01 onwards. Congenital anomalies were defined as having an International Classification of Diseases (ICD), Eighth Revision (2) (ICD-8) or ICD-9 (3) diagnosis in the range 740–759 or an ICD-10-SE (4) diagnosis in the range Q00–Q99 recorded in the Medical Birth Register.

Twins, siblings, and cousins were drawn from the same Swedish birth cohort (birth year 1973–2001) as the index individuals. Thus, for the analysis of "within-generation" co-aggregation among relative pairs, one pair may be included twice: first with A as exposure individual and B as outcome individual, then with B as exposure individual and A as outcome individual. A description of the birth cohort can be found in **Supplementary Table S4**.

However, the mothers, fathers, and aunts/uncles were allowed to have a birth year of ≥1933 and could be born either in or outside of Sweden. Thus, for the analysis of "between-generation" relative pairs, the child or niece/nephew was always treated as the exposure individual, and the parent or aunt/uncle as the outcome individual. A description of the birth year and age at end of follow-up (first emigration, death, or 2020-12-31) for different cohorts can be found in **Supplementary Table S6**.

To ensure our assumptions about the degree of common environment shared between different types of relatives would not be strongly violated, we excluded links to full and half-siblings when individuals were adopted away. And to ensure our assumptions about the degree of shared genetic variation between different types of relatives would not be strongly violated, we excluded links between aunts/uncles and nieces/nephews if the aunts/uncles were possible monozygotic twins with the parent of the niece/nephew (*i.e.*, aunt/uncle and parent with same birth date and sex, as coverage of the Swedish Twin Registry (5) and thus information about zygosity is incomplete for older individuals). We also excluded links between first cousins that were the children of possible monozygotic twins and excluded links between double first cousins (*i.e.*, cousins sharing all four grandparents), as these would also have a larger degree of shared genetic variation than expected.

To allow enough time for individuals to receive a diagnosis of the phenotypes investigated, we excluded individuals who died or emigrated from Sweden prior to age 18 years within each cohort of relative pairs for the familial co-aggregation and quantitative genetic analyses. Death or emigration before age 18 resulted in the exclusion of 18,829 and 101,376 and individuals from the birth cohort, respectively, leaving 2,665,478 individuals.

Phenotype ascertainment

Borderline personality disorder (BPD) was defined as ≥1 registered primary or secondary diagnosis of ICD-9 code 301.D or 301.J or ICD-10-SE code F60.3 in the Swedish National Patient Register (6,7) (NPR). The NPR contains discharge diagnoses from public inpatient care beginning in 1973, with near full coverage of diagnoses from inpatient care from 1987 onwards and diagnoses from specialist outpatient and emergency care from 2001 onwards. Information about primary and secondary discharge diagnoses, start/stop date of contact, and the department are routinely collected as a byproduct of the tax-funded universal healthcare system. The NPR does not contain information about primary care/general practitioner or private healthcare contacts. The ICD-8 was used from 1973 to 1986, ICD-9 from 1987 to 1996, and ICD-10-SE since 1997. In general, data from the NPR has been shown to have good to excellent validity (positive predictive value = 85–95%) for a range of conditions (6).

Similarly, other phenotypes were also defined as ≥1 registered primary or secondary discharge diagnosis of their respective ICD codes (**Supplementary Tables S1–S3**). For the ascertainment of all psychiatric phenotypes including BPD, we did not consider any diagnoses made before the age of 1 year, as these are likely due to human error and not a true diagnosis.

Within-individual and familial co-aggregation analysis

We used generalized estimating equations, with all phenotypes treated as lifetime diagnoses, to first estimate the within-individual association of BPD with other phenotypes (*i.e.*, odds of co-occurrence), adjusting for sex and birth year where applicable. Phenotypes were treated as lifetime diagnosis because previous studies have shown that there is often a considerable delay between symptom onset and official diagnosis for both psychiatric and somatic health conditions (8,9). Then, to describe familial co-aggregation, we calculated odds ratios for each BPD-phenotype association within each cohort of relative pairs, adjusting for sex, sex of relative, birth year, and birth year of relative where applicable. We used the R package drgee (10) (version 1.1.10) and calculated cluster-robust standard errors to account for the non-independence of family data. 268 additional father-child pairs were included in the analysis where the biological father was known but the biological mother was not known.

Covariate adjustment for different relative pairs

Odds ratio and hazard ratio estimates were adjusted for sex, sex of the relative, birth year, and birth year of the relative in the full sibling, half-sibling, aunt/uncle-niece/nephew, and first cousin pairs. For mother-child and father-child pairs, we only adjusted for sex, birth year, and birth year of the mother/father, as the sex of biological mothers is always female, and the sex of biological fathers is always male. For dizygotic twin pairs, we only adjusted for sex, sex of the twin, and birth year of the twin pair (instead of entering the birth year of each twin separately). For monozygotic twin pairs, we only adjusted for the sex of the twin pair and birth year of the twin pair (instead of entering the sex and birth year of each twin separately). Birth year was always entered as a natural cubic spline with five degrees of freedom using the *ns* function from the base R package splines (11) (version 4.3.2) to account for varying secular trends and age at peak incidence in diagnosis for each phenotype. For female-only phenotypes, only the sex of the exposure individual was adjusted for in the regression model.

An overview of the covariate adjustment for different relative pairs in the familial co-aggregation analysis is provided below (written in R formula style for the syntax drgee::gee(formula = Phenotype$_{\text{outcome}}$ ~ BPD$_{\text{exposure}}$ + …) and survival::coxph(formula = Surv(tstartAge, tstopAge, Phenotype$_{\text{outcome}}$ ~ BPD$_{\text{exposure}}$ + …)):

Within-individual:	sex + ns(byear, df=5)
Monozygotic twins:	sex$_{\text{twin pair}}$ + ns(byear$_{\text{twin pair}}$, df=5)
Dizygotic twins:	sex$_{\text{outcome}}$ + sex$_{\text{exposure}}$ + ns(byear$_{\text{twin pair}}$, df=5)
Full and half-siblings:	sex$_{\text{outcome}}$ + sex$_{\text{exposure}}$ + ns(byear$_{\text{outcome}}$, df=5) + ns(byear$_{\text{exposure}}$, df=5)
Mother-child:	sex$_{\text{child}}$ + ns(byear$_{\text{mother}}$, df=5) + ns(byear$_{\text{child}}$, df=5)
Father-child:	sex$_{\text{child}}$ + ns(byear$_{\text{father}}$, df=5) + ns(byear$_{\text{child}}$, df=5)
Aunt/uncle-niece/nephew:	sex$_{\text{aunt/uncle}}$ + sex$_{\text{niece/nephew}}$ + ns(byear$_{\text{aunt/uncle}}$, df=5) + ns(byear$_{\text{niece/nephew}}$, df=5)
Cousins:	sex$_{\text{outcome}}$ + sex$_{\text{exposure}}$ + ns(byear$_{\text{outcome}}$, df=5) + ns(byear$_{\text{exposure}}$, df=5)

Female-only phenotypes

The phenotypes polycystic ovary syndrome and sexual pain (**Table S2**) were only ascertained in individuals with female sex. Thus, for the within-individual analyses, association estimates were calculated using only females, and sex was not included as a covariate. And for the familial co-aggregation analyses, association estimates were calculated using only females as the outcome individuals and with only the sex of the exposure individual included as a covariate.

An overview of the covariate adjustment for different relative pairs in the familial co-aggregation analysis of female-only phenotypes is provided below:

Within-individual:	ns(byear, df=5)
Monozygotic twins:	ns(byear$_{\text{twin pair}}$, df=5)
Dizygotic twins:	sex$_{\text{exposure}}$ + ns(byear$_{\text{twin pair}}$, df=5)
Full and half-siblings:	sex$_{\text{exposure}}$ + ns(byear$_{\text{outcome}}$, df=5) + ns(byear$_{\text{exposure}}$, df=5)
Mother-child:	sex$_{\text{child}}$ + ns(byear$_{\text{mother}}$, df=5) + ns(byear$_{\text{child}}$, df=5)
Father-child:	sex$_{\text{child}}$ + ns(byear$_{\text{father}}$, df=5) + ns(byear$_{\text{child}}$, df=5)
Aunt/uncle-niece/nephew:	sex$_{\text{niece/nephew}}$ + ns(byear$_{\text{aunt/uncle}}$, df=5) + ns(byear$_{\text{niece/nephew}}$, df=5)
Cousins:	sex$_{\text{exposure}}$ + ns(byear$_{\text{outcome}}$, df=5) + ns(byear$_{\text{exposure}}$, df=5)

For the quantitative genetic analyses of female-only phenotypes, we selected siblings the same way as for the full population but only considered female same-sex relative pairs. As follows, we only adjusted for the birth year of each individual in the relative pair when modeling these phenotypes. The number of female full sibling pairs was 244,000, the number of female maternal half-sibling pairs was 35,512, and the number of female paternal half-sibling pairs was 35,257.

Definition of family clusters

Cluster-robust standard errors were calculated for the within-individual and between-relative association estimates in the familial co-aggregation analysis. Cluster definition varied between analyses. For mother-child pairs, clusters were defined by unique mothers (and likewise for father-child pairs). For aunt/uncle-niece/nephew pairs, clusters were defined by the unique maternal grandmothers of the nieces/nephews in the birth cohort (*i.e.*, the mothers of their aunts/uncles), as information about biological mothers was the most complete across generations. For first cousin pairs, clusters were also defined by unique maternal grandmothers. For within-individual associations and all other relative pairs (twins, full siblings, and half-siblings), clusters were defined by a family ID identifying all who shared at least one parent. That is, the family ID described larger clusters where individuals are joined to their siblings (twins and full and half-siblings) as well as all siblings (twins and full and half-siblings) of their half-siblings.

Quantitative genetic modeling

For quantitative genetic modeling, one unique sibling pair of each type (full siblings, maternal half-siblings, and paternal half-siblings) was selected from each family cluster using the following algorithm: 1) closest in age, 2) oldest, 3) random selection. We retained 765,171 unique full sibling, 91,475 maternal half-sibling, and 85,399 paternal half-sibling pairs for quantitative genetic modeling. Full siblings share on average 50% of their DNA identical by descent, while half-siblings share on average 25%.

We restricted quantitative genetic analyses to phenotypes with some evidence of association with BPD, which was defined as a within-individual tetrachoric correlation of ≥0.1. Within-individual tetrachoric correlations were calculated using the R package psych (12) (version 2.4.6.26) in the full birth cohort. In addition, to ensure that sufficient data was available, we only modeled phenotypes which had at least five sibling pairs discordant and concordant for BPD and the phenotype for each type of sibling pair (*i.e.*, full siblings, maternal half-siblings, and paternal half-siblings). That is, where there were at least 5 sibling pairs of each possible combination (using the phenotype depression as an example): BPD case + depression case, BPD control + depression case, BPD case + depression control, and BPD control + depression control. Based on these criteria, congenital hypothyroidism, cystic fibrosis, cerebral palsy, type 1 diabetes mellitus, autoimmune disease, and back, neck, and joint pain were excluded. In total, we performed quantitative genetic analysis for 38 phenotypes.

We first performed univariate structural equation modeling (SEM) for all phenotypes with and without adjustment for sex and birth year to guide future analytical decisions. We conducted SEM with relative pairs to gauge the contribution of shared additive genetic factors (A), common environmental factors (*i.e.*, factors that make members of the same family more similar) (C), and unique environmental factors (E) to observed associations (13). We did not fit reduced models, such as the AE model, to avoid potentially biased estimates (14). For univariate SEM, we did not constrain the variance components to be positive in the ACE model and used maximum likelihood for model fitting. On average, adjustment for sex and birth year (both centered on the sample mean) was found to produce more conservative heritability estimates and was deemed suitable for bivariate SEM. We observed a considerably lower-than-expected heritability (univariate A) and wide confidence intervals across all estimates for anorexia nervosa (**Supplementary Table S10**) (15). We also observed large negative estimates for proportion of phenotypic variance explained by common environmental factors (univariate C) and wide confidence intervals across all estimates for the following phenotypes: other eating disorder, other specific personality disorder, type 2 diabetes mellitus, and chronic body aches / fatigue. The confidence interval for the univariate C estimate included zero for 22 of the phenotypes, including BPD, which is in line with previous findings (16). The initial univariate heritability estimates were, however, not used for bivariate SEM.

We then performed bivariate SEM. Bivariate ACE models were fitted using the correlated factors approach (**Supplementary Figure S1**) and weighted least squares for model fitting. A more detailed explanation of the model can be found below:

Using BPD and depression (Dep) as an example, the modeled covariance matrix for a given sibling pair can be written as:

$$Cov\left(\begin{bmatrix} BPD_1 \\ Dep_1 \\ BPD_2 \\ Dep_2 \end{bmatrix}\right) =$$

$$\begin{bmatrix} a_{BPD}^2 + c_{BPD}^2 + e_{BPD}^2 & r_A a_{BPD} a_{Dep} + r_C c_{BPD} c_{Dep} + r_E e_{BPD} e_{Dep} & g \cdot a_{BPD}^2 + f \cdot c_{BPD}^2 & g \cdot r_A a_{BPD} a_{Dep} + f \cdot r_C c_{BPD} c_{Dep} \\ r_A a_{BPD} a_{Dep} + r_C c_{BPD} c_{Dep} + r_E e_{BPD} e_{Dep} & a_{Dep}^2 + c_{Dep}^2 + e_{Dep}^2 & g \cdot r_A a_{BPD} a_{Dep} + f \cdot r_C c_{BPD} c_{Dep} & g \cdot a_{Dep}^2 + f \cdot c_{Dep}^2 \\ g \cdot a_{BPD}^2 + f \cdot c_{BPD}^2 & g \cdot r_A a_{BPD} a_{Dep} + f \cdot r_C c_{BPD} c_{Dep} & a_{BPD}^2 + c_{BPD}^2 + e_{BPD}^2 & r_A a_{BPD} a_{Dep} + r_C c_{BPD} c_{Dep} + r_E e_{BPD} e_{Dep} \\ g \cdot r_A a_{BPD} a_{Dep} + f \cdot r_C c_{BPD} c_{Dep} & g \cdot a_{Dep}^2 + f \cdot c_{Dep}^2 & r_A a_{BPD} a_{Dep} + r_C c_{BPD} c_{Dep} + r_E e_{BPD} e_{Dep} & a_{Dep}^2 + c_{Dep}^2 + e_{Dep}^2 \end{bmatrix}$$

In this model, g = shared genetics identical by descent and f = assumed degree of shared familial environment of the sibling pair.

The model is fitted in the liability-threshold framework, which means that variances are fixed at 1. That is, $a_{BPD}^2 + c_{BPD}^2 + e_{BPD}^2 = 1$ and $a_{Dep}^2 + c_{Dep}^2 + e_{Dep}^2 = 1$.

Further estimates relevant for bivariate inferences (modeled or derived from the model):

- Genetic, shared and non-shared environmental correlations: r_A , r_C , r_E
- Phenotypic correlation: $r_{ph} = (r_A \cdot a_{BPD} \cdot a_{Dep}) + (r_C \cdot c_{BPD} \cdot c_{Dep}) + (r_E \cdot e_{BPD} \cdot e_{Dep})$
- Absolute explained phenotypic correlation by genetic $(r_A \cdot a_{BPD} \cdot a_{Dep})$, common environmental $(r_C \cdot c_{BPD} \cdot c_{Dep})$, and unique environmental factors $(r_E \cdot e_{BPD} \cdot e_{Dep})$.
- Relative explained phenotypic correlation by genetic $\left(\frac{r_A \cdot a_{BPD} \cdot a_{Dep}}{r_A \cdot a_{BPD} \cdot a_{Dep} + r_C \cdot c_{BPD} \cdot c_{Dep} + r_E \cdot e_{BPD} \cdot e_{Dep}}\right)$, common environmental $\left(\frac{r_A \cdot a_{BPD} \cdot a_{Dep} \cdot r_C \cdot c_{BPD} \cdot c_{Dep}}{r_A \cdot a_{BPD} \cdot a_{Dep} + r_C \cdot c_{BPD} \cdot c_{Dep} + r_E \cdot e_{BPD} \cdot e_{Dep}}\right)$, and unique environmental factors $\left(\frac{r_E \cdot e_{BPD} \cdot e_{Dep}}{r_A \cdot a_{BPD} \cdot a_{Dep} + r_C \cdot c_{BPD} \cdot c_{Dep} + r_E \cdot e_{BPD} \cdot e_{Dep}}\right)$,

Sex and birth year were centered in each subset of sibling pairs (full and maternal/paternal half-siblings) to aid weighted least squares model fitting as follows:

- $sex = sex - mean(sex)$
- $byear = \frac{byear - 1973}{28} - mean\left(\frac{byear - 1973}{28}\right)$

For three phenotypes—anorexia nervosa, other specific personality disorders, and assault/victimization—we encountered optimization problems with the bivariate models. Since these phenotypes all had an estimated univariate C that included zero in the confidence interval in the initial checks, we re-fitted the bivariate models with C fixed to 0 for these phenotypes and did not estimate the common environmental correlation (r_c) with BPD.

We reported estimates with Wald-type 95% confidence intervals, which are not bound within the parameter space (*e.g.*, -1.00 to 1.00 for correlations).

Sensitivity analysis

To assess the potential impact of differential follow-up time, we also treated BPD diagnosis as a time-varying exposure and calculated hazard ratios using Cox regression. In contrast to the main analysis, cohorts of relative pairs were not restricted to individuals alive and living in Sweden until age 18 years. Instead, individuals were followed from birth, first immigration to Sweden, or the start of the ICD-9 in 1987-01-01 (whichever came last), until the first emigration, first registered diagnosis of the phenotype of interest, death, or 2020-12-31 (whichever came first). As a result, outcome individuals with death or emigration before 1987 were excluded from the analysis. The sensitivity analysis used attained age as the underlying time scale and was conducted with the R package survival (17) (version 3.5.8).

Further notes

Statistical analyses were performed between 2024-02-01 and 2024-10-15 at Karolinska Institutet in Stockholm, Sweden using R(11) (version 4.3.2) and packages drgee (10) (version 1.1.10), OpenMx (18) (version 2.21.11), and survival (17) (version 3.5.8).

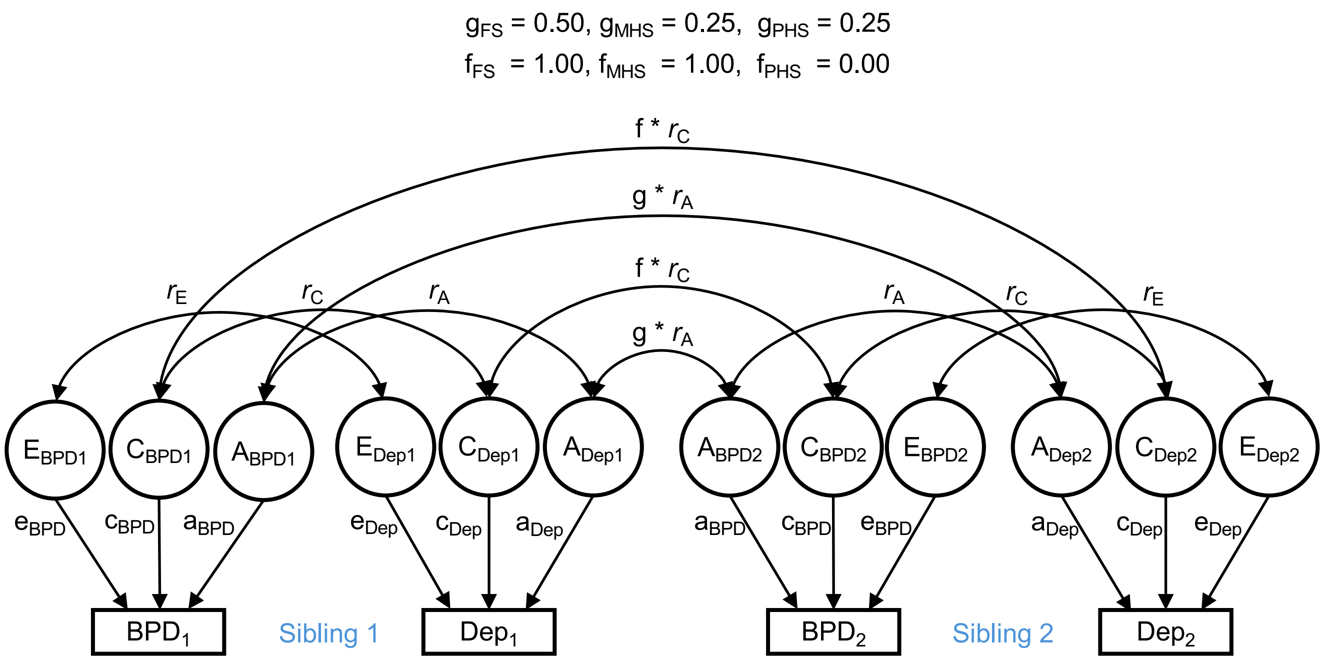

Supplementary Figure S1. Path diagram for bivariate structural equation model using different sibling pairs (correlated factors approach)
g = shared genetics identical by descent. f = assumed degree of shared familial environment. A = shared additive genetic factors. C = common/shared environmental factors. E = unique/non-shared environmental factors / measurement error. FS = full siblings. MHS = maternal half-siblings. PHS = paternal half-siblings. BPD = borderline personality disorder. Dep = depression. Paths/arrows for variances fixed to 1 and means model are omitted for easier reading.

Supplementary Table S1. ICD codes used to ascertain psychiatric phenotypes

Phenotype	ICD-8	ICD-9	ICD-10
**Alcohol use disorder**	291, 303	291, 303, 305.A	F10
**Other substance use disorder**	304, 971.03, 971.04	292, 304, 305 (excluding 305.A)	F11–F19 (excluding F17)
**Schizophrenia spectrum disorders**	295, 297, 298, 299 (excluding 295.50)	295, 297, 298 (excluding 295.F, 295.H, and 298.A)	F20–F29
**Bipolar disorders**	296 (excluding 296.20)	295.H, 296 (excluding 296.B)	F30–F31
**Depressive disorders**	296.20, 300.40	296.B, 298.A, 300.E, 311	F32, F33, F34.1–F34.9, F38, F39
**Anxiety disorders**	300.00, 300.20	300.A, 300.C	F40.0–F40.2, F41.0, F41.1
**Obsessive-compulsive disorder**	300.3	300.D	F42
**Acute stress reaction**		308, 309.A	F43.0
**Post-traumatic stress reaction**		309.B	F43.1
**Adjustment disorder**	307	309.X	F43.2, F43.8, F43.9
**Anorexia nervosa**		307.B	F50.0, F50.1
**Other eating disorder**		307.F	F50.2, F50.3, F50.9
**Borderline / Emotionally unstable personality disorder**		301.D, 301.J	F60.3
**Other specific personality disorder**	301.00–301.88	301.A–301.W (excluding 301.D and 301.J)	F60.0–F60.8, (excluding F60.3)
**Intellectual disability**	310–315	317–319	F70–F79
**Autism spectrum disorder**		299.A	F84.0, F84.1, F84.5, F84.8, F84.9
**Attention-deficit hyperactivity disorder**		314	F90
**Conduct disorder**		312	F91
**Childhood anxiety and emotional disorders**		313	F93
**Tic disorders**	306.2	307.C	F95

Supplementary Table S2. ICD codes used to ascertain somatic phenotypes

Phenotype	ICD-8	ICD-9	ICD-10
**Epilepsy**	345	345	G40, G41
**Cerebral palsy**	343.0	343	G80
**Migraine**	346.09	346.A, 346.B, 346.W, 346.X	G43
**Sleep disorder**	306.40	307.E, 780.F	F51, G25.8, G47
**Type 1 diabetes**	250 (age ≤30 years)	250 (age ≤30 years)	E10
**Type 2 diabetes**	250 (age >30 years)		
**Autoimmune disease**	034.1, 136.07, 242.00, 245.03, 269.10, 287.00, 287.10, 258.10, 390–392, 340, 354.01, 446.09, 446.30, 446.38, 446.40, 563.00, 563.10, 571.90, 580, 582, 694, 696.10, 696.19, 696.20–696.23, 704.00, 712.00, 712.10, 712.39, 716.00, 733.00, 734.00, 734.10	034.B, 136.B, 242.A, 245.C, 258.B, 287.A, 287.D, 340, 357.A, 358.A, 390–392, 446.A, 446.B, 446.F, 446.G, 555, 580, 582, 556, 571.F, 579.A, 694.A, 694.E, 694.F, 696, 710.A, 710.B, 710.C, 704.A, 710.D, 710.W, 714.A, 725	A38.9, D68.6, D69.0, D69.3, E05.0, E06.3, E31.0, G04, G13.1, G35, G61.0, G70.0, I00–I02, L10.0, L12.0, L13, L40, L63, K90.0, K50, K51, K74.3, M06, M30.0, M30.1, M31.1, M31.5, M32, M33.9, M34, M35.0, M35.2, M35.3, N00 N01, N03, N05, M30.3, M31.7, M35.1
**Congenital hypothyroidism**	243	243, 246.B	E03.0, E03.1
**Cystic fibrosis**			E84
**Anaphylaxis**	999.4	995.A, 995.B	T78.2, T78.3
**Asthma**	493	493	J45, J46
**Viral and/or bacterial infections**		001–005, 010–027, 008, 030–033, 034.B, 035–039, 041, 045–049, 050–068, 070–076, 077.A, 077.B, 077.C, 077.D, 077.E, 077.W, 077.X, 078A, 078.B, 078.D, 078.E, 078.F, 078.G, 078.H, 078.J, 078.W, 079, 080–083, 087, 091–098, 099.A, 099.B, 099C, 100–104, 320, 321.E, 321.H, 324–326, 382, 390, 391, 466.A, 466.B, 475, 478.B, 478.C, 480–482, 487, 510, 513, 540–542, 590, 595, 597.A, 599.A, 614–616, 646.F, 646.G, 680–686, 711.A, 711.E, 711.F, 790.H, 790.W	A00–A05, A15–A28, A30–A32, A34–A39, A40–A49, A51–A58, A65–A69, A70–A99, B00–B30, B33, B34, B95–B97, G00, G01, G02.0, G04.2, G05.0, G05.1, G06–G09, H66, H67.0, H67.1, I00, I01, J10–J12, J13–J15, J17.0, J17.1, J20.0–J20.2, J20.3–J20.7, J21.0, J36, J39.0, J39.1, J85, J86, K35–K37, L00–L09, M00, M01.0–M01.5, N10–N12, N30, N34.0, N39.0, N70–N79, O23
**Cardiovascular disease**	390–440, 444, 445, 450–453, 458	390–440, 444, 445	I00–I70, I73.0, I74, I75
**Polycystic ovary syndrome**[a]	(lifetime exclusion if 226.2, 253, 255, 759.5)	256.E (lifetime exclusion if 227.D, 253, 255, 611.G, 758.G)	E28.2 (lifetime exclusion if D35.2, E22, E24, E25, E27 N64.3, C74, O96)

Phenotype	ICD-8	ICD-9	ICD-10
**Sexual pain**[a]			F52.5, F52.6, N94
**Gastrointestinal problems**			K21, K58
**Persistent somatic fatigue and body aches**			M79.7, G93.3
**Back, neck & joint pain**			K07.6, M00–M29, M40–M59, R26

[a] Only ascertained for individuals with female sex.

Supplementary Table S3. ICD codes used to ascertain behavioral phenotypes and injuries

Phenotype	ICD-8	ICD-9	ICD-10
**Accidental poisoning**	E850–E877	E850–E869	X40–X49
**Transport-related accident**	E807–E846	E800–E849	V00–V99
**Fall-related accident**	E880–E887	E880–E888	W00–W19
**Traumatic brain injury**	800–804, 850–854	800–804, 850–854	S02.0, S02.1, S02.7–S02.9, S04.0, S06, S07.1
**Assault / victimization**	E960–E969	E960–E969	X85–X99, Y01–Y09
**Self-harm**	E950–E959, E980–E989	E950–E959, E980–E989	X60–X84, Y10–Y34, Y87.0, Y87.2
**Death by suicide**[a]	E950–E959, E980–E989	E950–E959, E980–E989	X60–X84, Y10–Y34, Y87.0, Y87.2

[a] Death by suicide was ascertained from the Swedish Cause of Death Register

Supplementary Table S4. Number of registered diagnoses for borderline personality disorder (BPD) cases in birth cohort

Number of BPD diagnoses per case	Frequency	(%)
1	2,150	8.9%
2	3,404	14.1%
3	1,031	4.3%
4	1,736	7.2%
5–9	3,587	16.0%
10–29	6,814	28.3%
30+	5,081	21.1%

Diagnosis of ICD-9 code 301.D or 301.J or ICD-10-SE code F60.3 in the Swedish National Health Register. Borderline personality disorder cases defined as ≥1 diagnosis. Percentages correspond to the column-wise sum.

Supplementary Table S5. Birth year and age at end of follow-up of different cohorts

	Birth cohort	Mothers	Fathers	Aunts/Uncles
**Birth year**				
Mean (SD)	1987 (8)	1958 (10)	1956 (10)	1957 (12)
Range	1973–2001	1933–1987	1933–1987	1933–2001
**Age at end of follow-up**				
Mean (SD)	32.9 (8.3)	59.9 (11.8)	61.4 (12.1)	60.6 (13.3)
Range[a]	0–48.0	0–88.0	0–88.0	0–88.0

SD = standard deviation.
[a] The maximum age is above the maximum possible value for each cohort due to rounding.

Supplementary Table S6. Familial co-aggregation results for psychiatric phenotypes with borderline personality disorder

Phenotype	Relative pair	Number of individuals or pairs for odds ratio[a]	Number of individuals unexposed to BPD for odds ratio		Number of individuals exposed to BPD for odds ratio		Adjusted odds ratio[b] [95%CI]	Number of individuals or pairs for hazard ratio[a]	Adjusted hazard ratio[b] [95%CI]
			Control	Case	Control	Case			
**Alcohol use disorder**	Within-indiv	2,665,478	2,532,236	109,169	16,293	7,780	11.8 [11.5, 12.2]	2,749,252	9.0 [8.6, 9.4]
	MZ twins	7,088	13,590	482	86	18	5.8 [3.3, 10.2]	7,200	7.2 [3.6, 14.7]
	DZ twins	13,276	25,397	965	178	12	1.7 [0.9, 3.1]	13,510	1.9 [0.8, 4.7]
	Full sibs	1,434,189	2,724,955	119,495	21,680	2,248	2.3 [2.2, 2.4]	1,497,592	2.1 [2.0, 2.3]
	Mat. half-sibs	243,717	441,273	37,204	8,000	957	1.5 [1.4, 1.6]	259,556	1.4 [1.2, 1.5]
	Pat. half-sibs	249,296	455,386	34,834	7,586	786	1.4 [1.3, 1.5]	267,971	1.3 [1.1, 1.5]
	Cousins	5,433,963	10,295,667	475,482	90,909	5,871	1.4 [1.3, 1.4]	5,693,457	1.4 [1.3, 1.4]
**Drug use disorder**	Within-indiv	2,665,478	2,564,240	77,165	14,963	9,110	26.3 [25.6, 27.1]	2,749,252	19.9 [19.2, 20.7]
	MZ twins	7,088	13,788	284	82	22	12.6 [7.3, 21.8]	7,200	19.0 [10.8, 33.5]
	DZ twins	13,276	25,779	583	175	15	3.3 [1.9, 5.9]	13,510	2.5 [1.0, 5.9]
	Full sibs	1,434,189	2,762,670	81,780	21,972	1,956	3.0 [2.8, 3.1]	1,497,592	2.9 [2.7, 3.1]
	Mat. half-sibs	243,717	445,933	32,544	8,004	953	1.7 [1.6, 1.8]	259,556	1.6 [1.5, 1.8]
	Pat. half-sibs	249,296	460,260	29,960	7,600	772	1.6 [1.5, 1.7]	267,971	1.5 [1.4, 1.7]
	Cousins	5,433,963	10,439,962	331,187	92,051	4,729	1.6 [1.6, 1.7]	5,693,457	1.6 [1.5, 1.7]
**Schizophrenia spectrum disorder**	Within-indiv	2,665,478	2,618,256	23,149	21,291	2,782	18.3 [17.5, 19.1]	2,749,106	16.7 [15.7, 17.7]
	MZ twins	7,088	13,969	103	94	10	15.0 [7.1, 31.7]	7,200	22.6 [10.2, 50.1]
	DZ twins	13,276	26,160	202	177	13	8.3 [4.6, 15.2]	13,510	9.8 [4.4, 21.9]
	Full sibs	1,434,189	2,818,050	26,400	23,380	548	2.5 [2.3, 2.7]	1,497,591	2.6 [2.3, 2.9]
	Mat. half-sibs	243,717	471,163	7,314	8,724	233	1.8 [1.5, 2.1]	259,556	1.7 [1.4, 2.1]
	Pat. half-sibs	249,296	483,198	7,022	8,189	183	1.6 [1.3, 1.8]	267,967	1.6 [1.3, 2.0]
	Cousins	5,433,963	10,670,959	100,190	95,495	1,285	1.4 [1.4, 1.5]	5,693,448	1.5 [1.3, 1.6]
**Bipolar disorder**	Within-indiv	2,665,478	2,609,007	32,398	17,682	6,391	23.5 [22.8, 24.3]	2,749,246	14.8 [14.2, 15.4]
	MZ twins	7,088	13,948	124	96	8	7.8 [3.8, 16.1]	7,200	NA
	DZ twins	13,276	26,081	281	180	10	5.1 [2.6, 9.8]	13,510	4.5 [1.8, 11.3]
	Full sibs	1,434,189	2,804,857	39,593	22,924	1,004	3.1 [2.8, 3.3]	1,497,592	2.9 [2.6, 3.2]
	Mat. half-sibs	243,717	467,293	11,184	8,626	331	1.6 [1.4, 1.9]	259,556	1.6 [1.4, 1.9]
	Pat. half-sibs	249,296	479,700	10,520	8,103	269	1.5 [1.4, 1.8]	267,971	1.5 [1.3, 1.8]
	Cousins	5,433,963	10,609,901	161,248	94,780	2,000	1.4 [1.3, 1.5]	5,693,457	1.4 [1.3, 1.4]
**Depressive disorder**	Within-indiv	2,665,478	2,422,554	218,851	5,641	18,432	30.6 [29.7, 31.6]	2,749,204	8.9 [8.5, 9.2]
	MZ twins	7,088	13,125	947	57	47	9.3 [6.1, 14.2]	7,200	6.7 [4.2, 10.9]
	DZ twins	13,276	24,466	1,896	157	33	2.7 [1.8, 4.1]	13,510	2.2 [1.3, 3.9]

Phenotype	Relative pair	Number of individuals or pairs for odds ratio[a]	Number of individuals unexposed to BPD for odds ratio		Number of individuals exposed to BPD for odds ratio		Adjusted odds ratio[b] [95%CI]	Number of individuals or pairs for hazard ratio[a]	Adjusted hazard ratio[b] [95%CI]
			Control	Case	Control	Case			
	Full sibs	1,434,189	2,602,492	241,958	19,301	4,627	2.6 [2.5, 2.7]	1,497,590	2.2 [2.1, 2.3]
	Mat. half-sibs	243,717	413,090	65,387	7,194	1,763	1.6 [1.5, 1.7]	259,556	1.4 [1.3, 1.6]
	Pat. half-sibs	249,296	428,413	61,807	7,008	1,364	1.4 [1.3, 1.5]	267,971	1.3 [1.2, 1.4]
	Cousins	5,433,963	9,817,313	953,836	85,210	11,570	1.4 [1.4, 1.4]	5,693,454	1.3 [1.2, 1.3]
**Anxiety disorder**	Within-indiv	2,665,478	2,541,663	99,742	14,742	9,331	13.2 [12.8, 13.6]	2,749,160	7.6 [7.3, 7.9]
	MZ twins	7,088	13,597	475	79	25	7.4 [4.6, 11.8]	7,200	5.5 [3.0, 10.1]
	DZ twins	13,276	25,481	881	171	19	3.2 [2.0, 5.2]	13,510	2.6 [1.3, 5.1]
	Full sibs	1,434,189	2,734,360	110,090	21,714	2,214	2.5 [2.4, 2.6]	1,497,587	2.3 [2.1, 2.4]
	Mat. half-sibs	243,717	448,361	30,116	8,115	842	1.6 [1.4, 1.7]	259,555	1.6 [1.4, 1.8]
	Pat. half-sibs	249,296	461,497	28,723	7,743	629	1.3 [1.2, 1.4]	267,971	1.3 [1.1, 1.4]
	Cousins	5,433,963	10,337,024	434,125	91,618	5,162	1.3 [1.3, 1.4]	5,693,452	1.3 [1.2, 1.3]
**Obsessive-compulsive disorder**	Within-indiv	2,665,478	2,611,742	29,663	21,309	2,764	10.0 [9.6, 10.4]	2,749,235	8.1 [7.6, 8.6]
	MZ twins	7,088	13,900	172	96	8	6.0 [2.9, 12.5]	7,200	7.8 [3.2, 19.3]
	DZ twins	13,276	NA	NA	NA	NA	NA	13,510	NA
	Full sibs	1,434,189	2,812,076	32,374	23,287	641	2.4 [2.2, 2.6]	1,497,592	2.3 [2.0, 2.5]
	Mat. half-sibs	243,717	471,045	7,432	8,752	205	1.5 [1.3, 1.7]	259,556	1.7 [1.4, 2.0]
	Pat. half-sibs	249,296	483,030	7,190	8,209	163	1.3 [1.1, 1.6]	267,971	1.3 [1.1, 1.7]
	Cousins	5,433,963	10,647,213	123,936	95,345	1,435	1.3 [1.2, 1.4]	5,693,457	1.2 [1.1, 1.3]
**Acute stress reaction**	Within-indiv	2,665,478	2,584,352	57,053	17,223	6,850	15.4 [14.9, 15.8]	2,748,535	9.8 [9.4, 10.2]
	MZ twins	7,088	13,837	235	82	22	13.1 [7.7, 22.1]	7,200	13.4 [7.7, 23.4]
	DZ twins	13,276	25,937	425	182	8	2.5 [1.3, 5.0]	13,510	NA
	Full sibs	1,434,189	2,780,478	63,972	22,500	1,428	2.7 [2.6, 2.9]	1,497,570	2.6 [2.4, 2.8]
	Mat. half-sibs	243,717	457,254	21,223	8,314	643	1.7 [1.5, 1.9]	259,544	1.7 [1.5, 1.9]
	Pat. half-sibs	249,296	470,571	19,649	7,927	445	1.4 [1.2, 1.5]	267,966	1.3 [1.2, 1.5]
	Cousins	5,433,963	10,507,897	263,252	93,478	3,302	1.4 [1.4, 1.5]	5,693,424	1.4 [1.3, 1.4]
**Post-traumatic stress disorder**	Within-indiv	2,665,478	2,619,803	21,602	19,153	4,920	22.2 [21.4, 23.0]	2,749,256	16.4 [15.7, 17.2]
	MZ twins	7,088	13,968	104	89	15	14.9 [7.6, 29.2]	7,200	13.5 [6.4, 28.5]
	DZ twins	13,276	26,150	212	183	7	5.5 [2.6, 11.8]	13,510	6.3 [2.6, 15.3]
	Full sibs	1,434,189	2,817,965	26,485	23,219	709	3.3 [3.1, 3.6]	1,497,592	3.3 [3.0, 3.6]
	Mat. half-sibs	243,717	468,931	9,546	8,635	322	1.9 [1.6, 2.1]	259,556	1.9 [1.6, 2.2]
	Pat. half-sibs	249,296	481,515	8,705	8,155	217	1.5 [1.3, 1.7]	267,971	1.7 [1.4, 2.0]
	Cousins	5,433,963	10,667,401	103,748	95,240	1,540	1.7 [1.6, 1.8]	5,693,457	1.6 [1.5, 1.7]
**Adjustment disorder**	Within-indiv	2,665,478	2,571,510	69,895	17,687	6,386	10.5 [10.2, 10.8]	2,748,983	6.3 [6.1, 6.6]
	MZ twins	7,088	13,758	314	88	16	5.4 [3.1, 9.6]	7,200	6.5 [3.7, 11.7]

Phenotype	Relative pair	Number of individuals or pairs for odds ratio[a]	Number of individuals unexposed to BPD for odds ratio		Number of individuals exposed to BPD for odds ratio		Adjusted odds ratio[b] [95%CI]	Number of individuals or pairs for hazard ratio[a]	Adjusted hazard ratio[b] [95%CI]
			Control	Case	Control	Case			
**Adjustment disorder**	DZ twins	13,276	25,797	565	180	10	2.4 [1.2, 4.6]	13,509	NA
	Full sibs	1,434,189	2,768,519	75,931	22,379	1,549	2.5 [2.4, 2.7]	1,497,573	2.2 [2.1, 2.4]
	Mat. half-sibs	243,717	456,644	21,833	8,368	589	1.5 [1.4, 1.6]	259,553	1.4 [1.3, 1.6]
	Pat. half-sibs	249,296	469,385	20,835	7,938	434	1.3 [1.2, 1.4]	267,969	1.2 [1.0, 1.4]
	Cousins	5,433,963	10,463,091	308,058	93,113	3,667	1.3 [1.3, 1.4]	5,693,449	1.3 [1.2, 1.4]
**Anorexia nervosa**	Within-indiv	2,665,478	2,627,314	14,091	22,455	1,618	8.1 [7.7, 8.6]	2,749,251	9.7 [8.8, 10.7]
	MZ twins	7,088	13,940	132	96	8	6.7 [3.1, 14.3]	7,200	NA
	DZ twins	13,276	NA	NA	NA	NA	NA	13,510	NA
	Full sibs	1,434,189	2,827,985	16,465	23,702	226	1.6 [1.4, 1.9]	1,497,592	1.7 [1.4, 2.2]
	Mat. half-sibs	243,717	475,822	2,655	8,884	73	1.5 [1.2, 1.8]	259,556	1.3 [0.9, 1.9]
	Pat. half-sibs	249,296	487,561	2,659	8,328	44	1.0 [0.7, 1.3]	267,971	1.2 [0.8, 1.8]
	Cousins	5,433,963	10,710,375	60,774	96,165	615	1.1 [1.0, 1.2]	5,693,457	1.2 [1.0, 1.4]
**Other eating disorder**	Within-indiv	2,665,478	2,612,578	28,827	19,689	4,384	12.4 [12.0, 12.9]	2,749,255	10.2 [9.6, 10.8]
	MZ twins	7,088	13,873	199	90	14	8.2 [4.5, 15.2]	7,200	13.1 [5.6, 30.4]
	DZ twins	13,276	25,995	367	181	9	4.1 [2.1, 8.1]	13,510	NA
	Full sibs	1,434,189	2,809,820	34,630	23,323	605	2.1 [2.0, 2.3]	1,497,592	2.1 [1.8, 2.4]
	Mat. half-sibs	243,717	471,366	7,111	8,765	192	1.5 [1.2, 1.7]	259,556	1.4 [1.1, 1.7]
	Pat. half-sibs	249,296	483,097	7,123	8,229	143	1.2 [1.0, 1.4]	267,971	1.2 [0.9, 1.5]
	Cousins	5,433,963	10,640,755	130,394	95,327	1,453	1.2 [1.2, 1.3]	5,693,457	1.1 [1.0, 1.2]
**Borderline personality disorder**	Within-indiv	2,665,478	NA	NA	NA	NA	NA	2,749,255	NA
	MZ twins	7,088	13,992	80	80	24	33.9 [16.6, 69.2]	7,200	27.4 [14.2, 52.8]
	DZ twins	13,276	26,178	184	184	6	4.8 [1.5, 15.6]	13,510	NA
	Full sibs	1,434,189	2,821,346	23,104	23,104	824	4.4 [4.0, 4.9]	1,497,592	4.2 [3.8, 4.7]
	Mat. half-sibs	243,717	469,836	8,641	8,641	316	2.0 [1.7, 2.4]	259,556	2.0 [1.7, 2.4]
	Pat. half-sibs	249,296	482,054	8,166	8,166	206	1.5 [1.2, 1.9]	267,971	1.5 [1.2, 1.8]
	Cousins	5,433,963	10,675,775	95,374	95,374	1,406	1.6 [1.5, 1.8]	5,693,457	1.6 [1.5, 1.7]
**Other specific personality disorder**	Within-indiv	2,665,478	2,633,667	7,738	21,515	2,558	43.9 [41.7, 46.2]	2,749,201	32.9 [30.8, 35.2]
	MZ twins	7,088	14,035	37	92	12	39.6 [18.3, 85.7]	7,200	46.9 [17.0, 129.6]
	DZ twins	13,276	NA	NA	NA	NA	NA	13,510	NA
	Full sibs	1,434,189	2,834,359	10,091	23,631	297	3.4 [3.0, 3.9]	1,497,592	3.5 [3.0, 4.1]
	Mat. half-sibs	243,717	474,981	3,496	8,871	86	1.3 [1.1, 1.6]	259,556	1.0 [0.7, 1.5]
	Pat. half-sibs	249,296	486,927	3,293	8,293	79	1.5 [1.2, 1.9]	267,971	1.6 [1.2, 2.3]
	Cousins	5,433,963	10,729,231	41,918	96,176	604	1.6 [1.5, 1.7]	5,693,451	1.6 [1.4, 1.8]

Phenotype	Relative pair	Number of individuals or pairs for odds ratio[a]	Number of individuals unexposed to BPD for odds ratio		Number of individuals exposed to BPD for odds ratio		Adjusted odds ratio[b] [95%CI]	Number of individuals or pairs for hazard ratio[a]	Adjusted hazard ratio[b] [95%CI]
			Control	Case	Control	Case			
**Intellectual disability**	Within-indiv	2,665,478	2,465,710	175,695	13,646	10,427	14.7 [14.3, 15.1]	2,747,466	14.6 [14.1, 15.0]
	MZ twins	7,088	13,404	668	80	24	9.6 [5.7, 16.2]	7,198	11.0 [5.7, 21.0]
	DZ twins	13,276	24,434	1,928	169	21	1.9 [1.2, 2.9]	13,508	1.8 [0.7, 4.3]
	Full sibs	1,434,189	2,668,902	175,548	20,755	3,173	2.5 [2.4, 2.6]	1,497,540	2.6 [2.5, 2.8]
	Mat. half-sibs	243,717	419,283	59,194	7,368	1,589	1.5 [1.4, 1.6]	259,546	1.5 [1.4, 1.6]
	Pat. half-sibs	249,296	435,622	54,598	7,075	1,297	1.5 [1.4, 1.6]	267,967	1.4 [1.2, 1.5]
	Cousins	5,433,963	10,067,829	703,320	87,835	8,945	1.5 [1.4, 1.5]	5,693,382	1.4 [1.4, 1.5]
**Autism spectrum disorder**	Within-indiv	2,665,478	2,588,150	53,255	21,285	2,788	8.7 [8.3, 9.1]	2,749,256	12.9 [12.3, 13.6]
	MZ twins	7,088	13,857	215	96	8	7.9 [3.5, 18.1]	7,200	12.3 [4.3, 34.7]
	DZ twins	13,276	25,803	559	176	14	4.1 [2.3, 7.2]	13,510	4.4 [1.8, 10.8]
	Full sibs	1,434,189	2,790,402	54,048	22,911	1,017	2.4 [2.2, 2.6]	1,497,592	2.5 [2.2, 2.7]
	Mat. half-sibs	243,717	462,605	15,872	8,476	481	1.7 [1.5, 1.8]	259,556	1.8 [1.6, 2.0]
	Pat. half-sibs	249,296	475,675	14,545	8,010	362	1.5 [1.3, 1.6]	267,971	1.4 [1.2, 1.6]
	Cousins	5,433,963	10,562,119	209,030	94,128	2,652	1.4 [1.4, 1.5]	5,693,457	1.4 [1.3, 1.5]
**Attention deficit hyperactivity disorder**	Within-indiv	2,665,478	2,512,917	128,488	14,929	9,144	15.6 [15.2, 16.1]	2,749,254	14.4 [13.9, 14.8]
	MZ twins	7,088	13,612	460	83	21	11.2 [6.5, 19.5]	7,200	12.8 [6.5, 25.1]
	DZ twins	13,276	25,070	1,292	176	14	1.8 [1.0, 3.0]	13,510	NA
	Full sibs	1,434,189	2,717,826	126,624	21,368	2,560	2.7 [2.5, 2.8]	1,497,592	2.7 [2.5, 2.8]
	Mat. half-sibs	243,717	431,169	47,308	7,619	1,338	1.6 [1.5, 1.7]	259,556	1.5 [1.4, 1.6]
	Pat. half-sibs	249,296	446,651	43,569	7,308	1,064	1.5 [1.4, 1.6]	267,971	1.4 [1.3, 1.5]
	Cousins	5,433,963	10,247,724	523,425	89,762	7,018	1.5 [1.5, 1.6]	5,693,457	1.5 [1.4, 1.5]
**Conduct disorder**	Within-indiv	2,665,478	2,629,165	12,240	22,812	1,261	15.5 [14.5, 16.4]	2,749,242	36.4 [31.0, 42.8]
	MZ twins	7,088	NA	NA	NA	NA	32.0 [10.1, 102.0]	7,200	NA
	DZ twins	13,276	NA	NA	NA	NA	NA	13,510	NA
	Full sibs	1,434,189	2,833,191	11,259	23,638	290	3.2 [2.8, 3.7]	1,497,591	4.0 [2.8, 5.6]
	Mat. half-sibs	243,717	472,753	5,724	8,785	172	1.6 [1.4, 1.9]	259,556	1.4 [0.9, 2.1]
	Pat. half-sibs	249,296	485,061	5,159	8,239	133	1.5 [1.3, 1.9]	267,971	1.6 [1.1, 2.4]
	Cousins	5,433,963	10,722,924	48,225	96,054	726	1.7 [1.6, 1.8]	5,693,455	1.8 [1.5, 2.2]
**Childhood anxiety and emotional disorders**	Within-indiv	2,665,478	2,638,550	2,855	23,772	301	11.8 [10.4, 13.4]	2,749,256	12.8 [8.5, 19.2]
	MZ twins	7,088	NA	NA	NA	NA	NA	7,200	NA
	DZ twins	13,276	NA	NA	NA	NA	NA	13,510	NA
	Full sibs	1,434,189	2,841,593	2,857	23,858	70	3.2 [2.5, 4.1]	1,497,592	3.3 [1.9, 5.8]
	Mat. half-sibs	243,717	477,512	965	8,920	37	1.9 [1.4, 2.7]	259,556	2.0 [1.1, 3.6]

Phenotype	Relative pair	Number of individuals or pairs for odds ratio[a]	Number of individuals unexposed to BPD for odds ratio		Number of individuals exposed to BPD for odds ratio		Adjusted odds ratio[b] [95%CI]	Number of individuals or pairs for hazard ratio[a]	Adjusted hazard ratio[b] [95%CI]
			Control	Case	Control	Case			
	Pat. half-sibs	249,296	489,314	906	8,347	25	1.6 [1.1, 2.3]	267,971	1.8 [1.0, 3.4]
	Cousins	5,433,963	10,759,050	12,099	96,639	141	1.3 [1.1, 1.5]	5,693,457	1.4 [1.0, 1.9]
**Tic disorder**	Within-indiv	2,665,478	2,633,249	8,156	23,857	216	5.5 [4.8, 6.3]	2,749,208	8.9 [6.9, 11.3]
	MZ twins	7,088	NA	NA	NA	NA	NA	7,200	NA
	DZ twins	13,276	NA	NA	NA	NA	NA	13,510	NA
	Full sibs	1,434,189	2,837,049	7,401	23,787	141	2.4 [2.0, 2.8]	1,497,592	2.4 [1.7, 3.4]
	Mat. half-sibs	243,717	476,180	2,297	8,883	74	1.8 [1.4, 2.2]	259,556	1.8 [1.2, 2.6]
	Pat. half-sibs	249,296	488,011	2,209	8,323	49	1.2 [0.9, 1.7]	267,971	1.2 [0.7, 1.9]
	Cousins	5,433,963	10,741,485	29,664	96,415	365	1.4 [1.2, 1.6]	5,693,453	1.4 [1.1, 1.7]

95% CI = 95% Wald-type confidence interval. NA = not available (indicates that there were too few observations to estimate the association). Within-indiv = within-individual association. MZ twins = monozygotic twins. DZ twins = dizygotic twins. Mat. = maternal. Pat. = paternal. Sibs = siblings.

[a] Number of unique individuals (for within-individual analyses) or number of unique ways of combining pairs (for relative pair analyses, *i.e.*, a pair may be included twice, first with A as outcome individual and B as exposure individual, then with B as outcome individual and A as exposure individual).

[b] Adjusted for sex, sex of relative, birth year, and birth year of relative where applicable.

Supplementary Table S7. Familial co-aggregation results for somatic phenotypes with borderline personality disorder

Phenotype	Relative pair	Number of individuals or pairs for odds ratio[a]	Number of individuals unexposed to BPD for odds ratio		Number of individuals exposed to BPD for odds ratio		Adjusted odds ratio[b] [95%CI]	Number of individuals or pairs for hazard ratio[a]	Adjusted hazard ratio[b] [95%CI]
			Control	Case	Control	Case			
**Epilepsy**	Within-indiv	2,665,478	2,602,338	39,067	23,015	1,058	3.1 [2.9, 3.3]	2,746,194	6.1 [5.5, 6.7]
	MZ twins	7,088	NA	NA	NA	NA	NA	7,196	NA
	DZ twins	13,276	25,927	435	185	5	1.6 [0.7, 4.0]	13,510	NA
	Full sibs	1,434,189	2,803,346	41,104	23,441	487	1.4 [1.3, 1.6]	1,497,557	1.6 [1.3, 1.9]
	Mothers	2,632,129	2,568,600	39,955	23,004	570	1.6 [1.5, 1.7]	2,687,989	1.6 [1.4, 1.9]
	Fathers	2,602,867	2,530,556	49,124	22,541	646	1.5 [1.4, 1.6]	2,639,444	1.3 [1.1, 1.5]
	Mat. half-sibs	243,717	469,244	9,233	8,748	209	1.2 [1.0, 1.4]	259,549	1.4 [1.0, 1.8]
	Pat. half-sibs	249,296	481,387	8,833	8,197	175	1.2 [1.0, 1.4]	267,967	0.9 [0.7, 1.3]
	Aunts/uncles	8,074,278	7,828,902	174,729	68,829	1,818	1.2 [1.1, 1.3]	8,054,069	1.1 [1.0, 1.2]
	Cousins	5,433,963	10,609,406	161,743	95,113	1,667	1.1 [1.1, 1.2]	5,693,342	1.1 [0.9, 1.2]
**Cerebral palsy**	Within-indiv	2,665,478	2,634,561	6,844	24,015	58	1.0 [0.8, 1.3]	2,747,587	NA
	MZ twins	7,088	NA	NA	NA	NA	NA	7,200	NA
	DZ twins	13,276	NA	NA	NA	NA	NA	13,510	NA
	Full sibs	1,434,189	2,837,943	6,507	23,864	64	1.2 [0.9, 1.5]	1,497,570	NA
	Mothers	2,632,129	2,606,687	1,868	23,549	25	1.5 [1.0, 2.2]	2,694,161	1.3 [0.5, 3.1]
	Fathers	2,602,867	2,577,776	1,904	23,152	35	2.0 [1.4, 2.8]	2,644,855	1.5 [0.7, 3.2]
	Mat. half-sibs	243,717	477,035	1,442	8,933	24	0.9 [0.6, 1.4]	259,543	NA
	Pat. half-sibs	249,296	488,758	1,462	8,344	28	1.1 [0.8, 1.6]	267,968	NA
	Aunts/uncles	8,074,278	7,989,565	14,066	70,522	125	1.0 [0.8, 1.2]	8,079,672	0.9 [0.6, 1.4]
	Cousins	5,433,963	10,743,230	27,919	96,500	280	1.1 [1.0, 1.3]	5,693,387	1.0 [0.6, 1.6]
**Migraine**	Within-indiv	2,665,478	2,561,890	79,515	22,258	1,815	2.0 [1.9, 2.1]	2,748,993	2.0 [1.9, 2.2]
	MZ twins	7,088	13,732	340	93	11	3.6 [1.8, 7.1]	7,200	4.2 [1.8, 10.0]
	DZ twins	13,276	25,673	689	180	10	2.1 [1.1, 4.1]	13,510	NA
	Full sibs	1,434,189	2,761,024	83,426	22,951	977	1.4 [1.3, 1.5]	1,497,591	1.4 [1.3, 1.5]
	Mothers	2,632,129	2,507,582	100,973	22,397	1,177	1.3 [1.2, 1.4]	2,691,368	1.1 [1.0, 1.3]
	Fathers	2,602,867	2,546,550	33,130	22,840	347	1.2 [1.1, 1.3]	2,643,638	1.0 [0.9, 1.3]
	Mat. half-sibs	243,717	461,469	17,008	8,561	396	1.3 [1.1, 1.4]	259,554	1.2 [1.0, 1.4]
	Pat. half-sibs	249,296	473,320	16,900	8,079	293	1.0 [0.9, 1.2]	267,971	1.0 [0.9, 1.2]
	Aunts/uncles	8,074,278	7,821,094	182,537	68,834	1,813	1.1 [1.1, 1.2]	8,077,653	1.0 [0.9, 1.1]
	Cousins	5,433,963	10,439,469	331,680	93,481	3,299	1.1 [1.1, 1.2]	5,693,450	1.0 [1.0, 1.1]
**Sleep disorder**	Within-indiv	2,665,478	2,559,326	82,079	19,147	4,926	9.0 [8.7, 9.3]	2,749,116	7.7 [7.4, 8.0]
	MZ twins	7,088	13,768	304	92	12	5.9 [3.0, 11.6]	7,200	4.8 [2.0, 11.8]

Phenotype	Relative pair	Number of individuals or pairs for odds ratio[a]	Number of individuals unexposed to BPD for odds ratio		Number of individuals exposed to BPD for odds ratio		Adjusted odds ratio[b] [95%CI]	Number of individuals or pairs for hazard ratio[a]	Adjusted hazard ratio[b] [95%CI]
			Control	Case	Control	Case			
	DZ twins	13,276	25,605	757	179	11	2.2 [1.2, 4.0]	13,510	2.9 [1.3, 6.1]
	Full sibs	1,434,189	2,759,719	84,731	22,522	1,406	2.0 [1.9, 2.2]	1,497,590	2.0 [1.9, 2.2]
	Mothers	2,632,129	2,489,406	119,149	21,806	1,768	1.7 [1.6, 1.7]	2,694,486	1.6 [1.5, 1.7]
	Fathers	2,602,867	2,357,593	222,087	20,798	2,389	1.2 [1.1, 1.3]	2,645,156	1.1 [1.1, 1.2]
	Mat. half-sibs	243,717	455,326	23,151	8,328	629	1.5 [1.4, 1.6]	259,556	1.6 [1.4, 1.7]
	Pat. half-sibs	249,296	467,699	22,521	7,872	500	1.3 [1.2, 1.5]	267,971	1.3 [1.1, 1.5]
	Aunts/uncles	8,074,278	7,502,095	501,536	65,708	4,939	1.1 [1.1, 1.2]	8,084,464	1.0 [1.0, 1.1]
	Cousins	5,433,963	10,427,303	343,846	92,629	4,151	1.4 [1.3, 1.4]	5,693,454	1.3 [1.2, 1.4]
**Type 1 diabetes mellitus**	Within-indiv	2,665,478	2,614,846	26,559	23,635	438	2.0 [1.8, 2.2]	2,747,351	1.9 [1.6, 2.4]
	MZ twins	7,088	NA	NA	NA	NA	NA	7,198	NA
	DZ twins	13,276	NA	NA	NA	NA	NA	13,508	NA
	Full sibs	1,434,189	2,816,392	28,058	23,632	296	1.3 [1.1, 1.4]	1,497,555	1.1 [0.8, 1.4]
	Mothers	2,632,129	2,567,812	40,743	23,067	507	1.4 [1.3, 1.5]	2,685,471	1.2 [1.0, 1.5]
	Fathers	2,602,867	2,513,520	66,160	22,471	716	1.2 [1.1, 1.3]	2,638,745	1.0 [0.8, 1.2]
	Mat. half-sibs	243,717	473,623	4,854	8,869	88	1.0 [0.8, 1.2]	259,551	1.0 [0.7, 1.5]
	Pat. half-sibs	249,296	485,418	4,802	8,259	113	1.3 [1.1, 1.6]	267,970	1.1 [0.8, 1.6]
	Aunts/uncles	8,074,278	7,821,325	182,306	68,829	1,818	1.2 [1.1, 1.2]	8,058,295	1.0 [0.9, 1.1]
	Cousins	5,433,963	10,662,055	109,094	95,714	1,066	1.1 [1.0, 1.2]	5,693,390	1.1 [1.0, 1.3]
**Type 2 diabetes mellitus**	Within-indiv	2,665,478	2,628,119	13,286	23,615	458	4.2 [3.8, 4.6]	2,749,256	4.0 [3.6, 4.5]
	MZ twins	7,088	NA	NA	NA	NA	NA	7,200	NA
	DZ twins	13,276	NA	NA	NA	NA	NA	13,510	NA
	Full sibs	1,434,189	2,831,389	13,061	23,729	199	1.8 [1.5, 2.1]	1,497,592	1.7 [1.4, 2.1]
	Mothers	2,632,129	2,476,259	132,296	21,970	1,604	1.4 [1.3, 1.5]	2,689,044	1.4 [1.3, 1.5]
	Fathers	2,602,867	2,335,507	244,173	20,554	2,633	1.2 [1.2, 1.3]	2,639,907	1.2 [1.2, 1.3]
	Mat. half-sibs	243,717	475,302	3,175	8,890	67	1.1 [0.9, 1.4]	259,556	1.0 [0.7, 1.4]
	Pat. half-sibs	249,296	487,144	3,076	8,314	58	1.1 [0.9, 1.5]	267,971	1.0 [0.7, 1.5]
	Aunts/uncles	8,074,278	7,393,897	609,734	64,634	6,013	1.2 [1.1, 1.2]	8,064,639	1.1 [1.1, 1.2]
	Cousins	5,433,963	10,713,883	57,266	96,163	617	1.2 [1.1, 1.3]	5,693,457	1.2 [1.1, 1.3]
**Autoimmune disease**	Within-indiv	2,665,478	2,480,468	160,937	21,694	2,379	1.5 [1.4, 1.5]	2,743,985	1.4 [1.4, 1.5]
	MZ twins	7,088	13,213	859	93	11	1.5 [0.8, 2.8]	7,197	2.3 [1.1, 4.7]
	DZ twins	13,276	24,880	1,482	183	7	0.6 [0.3, 1.3]	13,510	NA
	Full sibs	1,434,189	2,672,262	172,188	22,257	1,671	1.2 [1.1, 1.2]	1,497,517	1.1 [1.0, 1.2]
	Mothers	2,632,129	2,311,255	297,300	20,391	3,183	1.2 [1.2, 1.3]	2,673,535	1.1 [1.0, 1.2]
	Fathers	2,602,867	2,372,851	206,829	21,207	1,980	1.1 [1.0, 1.1]	2,624,545	1.0 [0.9, 1.1]
	Mat. half-sibs	243,717	447,861	30,616	8,361	596	1.0 [1.0, 1.1]	259,546	1.0 [0.9, 1.2]

Phenotype	Relative pair	Number of individuals or pairs for odds ratio[a]	Number of individuals unexposed to BPD for odds ratio		Number of individuals exposed to BPD for odds ratio		Adjusted odds ratio[b] [95%CI]	Number of individuals or pairs for hazard ratio[a]	Adjusted hazard ratio[b] [95%CI]
			Control	Case	Control	Case			
	Pat. half-sibs	249,296	459,049	31,171	7,816	556	1.1 [1.0, 1.2]	267,960	1.0 [0.9, 1.2]
	Aunts/uncles	8,074,278	7,200,964	802,667	63,175	7,472	1.1 [1.0, 1.1]	8,011,447	1.0 [0.9, 1.0]
	Cousins	5,433,963	10,100,561	670,588	90,421	6,359	1.1 [1.0, 1.1]	5,693,233	1.0 [0.9, 1.0]
**Congenital hypothyroidism**	Within-indiv	2,665,478	2,639,791	1,614	24,038	35	1.8 [1.3, 2.5]	2,749,078	2.8 [1.3, 5.9]
	MZ twins	7,088	NA	NA	NA	NA	NA	7,200	NA
	DZ twins	13,276	NA	NA	NA	NA	NA	13,510	NA
	Full sibs	1,434,189	2,842,725	1,725	23,914	14	1.0 [0.6, 1.7]	1,497,587	NA
	Mothers	2,632,129	2,606,927	1,628	23,551	23	1.6 [1.0, 2.4]	2,694,550	NA
	Fathers	2,602,867	2,579,287	393	23,180	7	2.0 [0.9, 4.2]	2,645,228	NA
	Mat. half-sibs	243,717	478,133	344	8,952	5	0.8 [0.3, 2.2]	259,556	NA
	Pat. half-sibs	249,296	489,903	317	8,366	6	1.2 [0.5, 2.6]	267,971	NA
	Aunts/uncles	8,074,278	8,000,193	3,438	70,610	37	1.2 [0.9, 1.7]	8,084,657	0.6 [0.2, 1.4]
	Cousins	5,433,963	10,764,590	6,559	96,714	66	1.1 [0.8, 1.4]	5,693,452	0.7 [0.4, 1.4]
**Cystic fibrosis**	Within-indiv	2,665,478	2,640,829	576	24,068	5	1.0 [0.4, 2.3]	2,749,256	NA
	MZ twins	7,088	NA	NA	NA	NA	NA	7,200	NA
	DZ twins	13,276	NA	NA	NA	NA	NA	13,510	NA
	Full sibs	1,434,189	2,843,840	610	23,923	5	1.0 [0.4, 2.7]	1,497,592	NA
	Mothers	2,632,129	NA	NA	NA	NA	NA	2,694,686	NA
	Fathers	2,602,867	NA	NA	NA	NA	NA	2,645,306	NA
	Mat. half-sibs	243,717	NA	NA	NA	NA	NA	259,556	NA
	Pat. half-sibs	249,296	NA	NA	NA	NA	NA	267,971	NA
	Aunts/uncles	8,074,278	8,002,433	1,198	70,636	11	1.1 [0.6, 1.9]	8,085,079	NA
	Cousins	5,433,963	10,768,706	2,443	96,761	19	0.9 [0.5, 1.4]	5,693,457	NA
**Anaphylaxis**	Within-indiv	2,665,478	2,623,410	17,995	23,706	367	2.0 [1.8, 2.3]	2,749,135	2.4 [2.0, 2.8]
	MZ twins	7,088	NA	NA	NA	NA	NA	7,200	NA
	DZ twins	13,276	NA	NA	NA	NA	NA	13,510	NA
	Full sibs	1,434,189	2,825,713	18,737	23,717	211	1.3 [1.2, 1.5]	1,497,591	1.2 [1.0, 1.5]
	Mothers	2,632,129	2,586,364	22,191	23,319	255	1.3 [1.1, 1.5]	2,694,139	1.1 [0.9, 1.4]
	Fathers	2,602,867	2,559,446	20,234	22,990	197	1.1 [0.9, 1.2]	2,644,801	1.2 [1.0, 1.5]
	Mat. half-sibs	243,717	474,963	3,514	8,884	73	1.1 [0.9, 1.4]	259,556	1.0 [0.7, 1.5]
	Pat. half-sibs	249,296	486,441	3,779	8,297	75	1.2 [0.9, 1.5]	267,971	1.3 [0.9, 1.8]
	Aunts/uncles	8,074,278	7,942,597	61,034	70,106	541	1.0 [0.9, 1.1]	8,083,508	1.0 [0.9, 1.1]
	Cousins	5,433,963	10,698,762	72,387	96,081	699	1.1 [1.0, 1.2]	5,693,453	1.1 [1.0, 1.2]
**Asthma**	Within-indiv	2,665,478	2,433,169	208,236	20,717	3,356	2.1 [2.0, 2.1]	2,742,448	3.1 [2.9, 3.3]
	MZ twins	7,088	12,887	1,185	90	14	2.2 [1.1, 4.2]	7,195	4.1 [1.8, 9.4]

Phenotype	Relative pair	Number of individuals or pairs for odds ratio[a]	Number of individuals unexposed to BPD for odds ratio		Number of individuals exposed to BPD for odds ratio		Adjusted odds ratio[b] [95%CI]	Number of individuals or pairs for hazard ratio[a]	Adjusted hazard ratio[b] [95%CI]
			Control	Case	Control	Case			
	DZ twins	13,276	23,719	2,643	164	26	1.7 [1.1, 2.6]	13,509	NA
	Full sibs	1,434,189	2,635,354	209,096	21,695	2,233	1.3 [1.3, 1.4]	1,497,420	1.2 [1.1, 1.3]
	Mothers	2,632,129	2,461,142	147,413	21,533	2,041	1.6 [1.5, 1.6]	2,682,449	1.4 [1.3, 1.5]
	Fathers	2,602,867	2,477,466	102,214	22,005	1,182	1.3 [1.2, 1.4]	2,634,553	1.2 [1.1, 1.3]
	Mat. half-sibs	243,717	433,257	45,220	7,993	964	1.1 [1.1, 1.2]	259,527	0.9 [0.8, 1.0]
	Pat. half-sibs	249,296	445,711	44,509	7,490	882	1.2 [1.1, 1.3]	267,945	0.9 [0.8, 1.1]
	Aunts/uncles	8,074,278	7,625,774	377,857	66,689	3,958	1.2 [1.2, 1.2]	8,049,900	1.1 [1.0, 1.1]
	Cousins	5,433,963	9,949,095	822,054	88,588	8,192	1.1 [1.1, 1.2]	5,693,128	0.9 [0.9, 1.0]
**Infections**	Within-indiv	2,665,478	1,632,268	1,009,137	8,272	15,801	2.5 [2.5, 2.6]	2,749,039	2.3 [2.2, 2.4]
	MZ twins	7,088	8,726	5,346	48	56	1.7 [1.2, 2.5]	7,199	1.4 [0.9, 2.3]
	DZ twins	13,276	16,218	10,144	111	79	1.2 [0.9, 1.6]	13,510	1.2 [0.8, 1.9]
	Full sibs	1,434,189	1,778,929	1,065,521	13,442	10,486	1.3 [1.3, 1.3]	1,497,589	1.2 [1.2, 1.3]
	Mothers	2,632,129	1,636,435	972,120	12,847	10,727	1.4 [1.4, 1.5]	2,694,102	1.2 [1.2, 1.3]
	Fathers	2,602,867	1,842,615	737,065	15,546	7,641	1.2 [1.2, 1.3]	2,644,961	1.2 [1.1, 1.2]
	Mat. half-sibs	243,717	270,942	207,535	4,832	4,125	1.1 [1.1, 1.2]	259,555	1.0 [0.9, 1.0]
	Pat. half-sibs	249,296	280,930	209,290	4,574	3,798	1.1 [1.1, 1.2]	267,971	1.0 [1.0, 1.1]
	Aunts/uncles	8,074,278	5,405,712	2,597,919	45,781	24,866	1.1 [1.1, 1.2]	8,084,036	1.1 [1.0, 1.1]
	Cousins	5,433,963	6,710,220	4,060,929	58,146	38,634	1.1 [1.1, 1.1]	5,693,453	1.0 [1.0, 1.0]
**Cardiovascular disease**	Within-indiv	2,665,478	2,527,448	113,957	21,965	2,108	2.3 [2.2, 2.4]	2,747,374	2.5 [2.3, 2.6]
	MZ twins	7,088	13,472	600	92	12	3.0 [1.6, 5.6]	7,199	3.1 [1.5, 6.4]
	DZ twins	13,276	25,423	939	178	12	1.7 [1.0, 3.2]	13,510	2.0 [0.9, 4.1]
	Full sibs	1,434,189	2,728,533	115,917	22,718	1,210	1.2 [1.2, 1.3]	1,497,551	1.2 [1.1, 1.3]
	Mothers	2,632,129	1,934,425	674,130	16,405	7,169	1.3 [1.3, 1.4]	2,675,702	1.2 [1.1, 1.2]
	Fathers	2,602,867	1,610,644	969,036	13,818	9,369	1.1 [1.1, 1.2]	2,617,327	1.1 [1.1, 1.1]
	Mat. half-sibs	243,717	455,578	22,899	8,513	444	1.0 [0.9, 1.2]	259,550	1.1 [1.0, 1.3]
	Pat. half-sibs	249,296	466,737	23,483	7,935	437	1.1 [1.0, 1.3]	267,967	1.1 [1.0, 1.3]
	Aunts/uncles	8,074,278	5,371,674	2,631,957	46,709	23,938	1.1 [1.1, 1.1]	7,999,165	1.1 [1.0, 1.1]
	Cousins	5,433,963	10,292,206	478,943	92,006	4,774	1.1 [1.1, 1.2]	5,693,395	1.1 [1.0, 1.1]
	Within-indiv	1,301,755	1,251,935	29,159	19,757	904	1.8 [1.7, 1.9]	1,342,288	1.6 [1.5, 1.7]
	MZ twins	3,895	NA	NA	NA	NA	NA	3,960	NA

Phenotype	Relative pair	Number of individuals or pairs for odds ratio[a]	Number of individuals unexposed to BPD for odds ratio		Number of individuals exposed to BPD for odds ratio		Adjusted odds ratio[b] [95%CI]	Number of individuals or pairs for hazard ratio[a]	Adjusted hazard ratio[b] [95%CI]
			Control	Case	Control	Case			
**Polycystic ovary syndrome**	DZ twins	10,103	NA	NA	NA	NA	NA	10,286	NA
	Full sibs	1,056,825	1,351,296	34,058	11,276	342	1.2 [1.0, 1.3]	1,100,607	1.1 [0.9, 1.2]
	Mothers	2,632,129	2,602,718	5,837	23,513	61	1.2 [1.0, 1.6]	2,694,686	NA
	Fathers	0	0	0	0	0	NA	0	NA
	Mat. half-sibs	180,623	229,044	5,808	4,348	117	1.1 [0.9, 1.3]	190,764	1.1 [0.9, 1.4]
	Pat. half-sibs	184,844	234,824	5,923	4,011	107	1.1 [0.9, 1.3]	197,410	0.9 [0.7, 1.2]
	Aunts/uncles	3,935,614	3,888,772	12,433	34,285	124	1.2 [1.0, 1.4]	3,933,945	1.0 [0.6, 1.6]
	Cousins	4,011,435	5,139,413	121,896	46,053	1,261	1.1 [1.1, 1.2]	4,185,293	1.0 [1.0, 1.1]
**Sexual pain**	Within-indiv	1,301,755	1,147,673	133,421	15,900	4,761	2.5 [2.4, 2.5]	1,342,288	2.1 [2.0, 2.2]
	MZ twins	3,895	7,055	644	71	20	2.8 [1.7, 4.6]	3,960	1.8 [0.9, 3.7]
	DZ twins	10,103	12,176	1,161	78	11	1.4 [0.7, 2.6]	10,286	1.2 [0.5, 2.8]
	Full sibs	1,056,825	1,238,798	146,556	9,873	1,745	1.5 [1.4, 1.6]	1,100,607	1.3 [1.2, 1.4]
	Mothers	2,632,129	2,469,869	138,686	21,937	1,637	1.3 [1.2, 1.4]	2,694,677	1.0 [0.8, 1.1]
	Fathers	0	0	0	0	0	NA	0	NA
	Mat. half-sibs	180,623	204,811	30,041	3,788	677	1.2 [1.1, 1.4]	190,764	1.1 [1.0, 1.3]
	Pat. half-sibs	184,844	210,019	30,728	3,527	591	1.2 [1.1, 1.3]	197,410	1.2 [1.1, 1.3]
	Aunts/uncles	3,935,614	3,717,670	183,535	32,592	1,817	1.1 [1.1, 1.2]	3,933,935	0.8 [0.8, 0.9]
	Cousins	4,011,435	4,690,909	570,400	41,589	5,725	1.1 [1.1, 1.2]	4,185,293	1.1 [1.0, 1.1]
**Gastrointesti nal pain**	Within-indiv	2,665,478	2,567,715	73,690	22,124	1,949	2.6 [2.4, 2.7]	2,749,255	2.4 [2.3, 2.6]
	MZ twins	7,088	13,738	334	94	10	3.5 [1.8, 6.6]	7,200	NA
	DZ twins	13,276	25,751	611	178	12	2.7 [1.5, 4.9]	13,510	3.3 [1.5, 7.0]
	Full sibs	1,434,189	2,767,025	77,425	22,995	933	1.4 [1.3, 1.5]	1,497,592	1.3 [1.2, 1.5]
	Mothers	2,632,129	2,451,964	156,591	21,762	1,812	1.3 [1.2, 1.4]	2,694,684	1.2 [1.1, 1.3]
	Fathers	2,602,867	2,447,594	132,086	21,842	1,345	1.1 [1.1, 1.2]	2,645,294	1.0 [0.9, 1.1]
	Mat. half-sibs	243,717	463,628	14,849	8,647	310	1.1 [1.0, 1.3]	259,556	1.1 [0.9, 1.3]
	Pat. half-sibs	249,296	474,862	15,358	8,073	299	1.2 [1.0, 1.3]	267,971	1.1 [0.9, 1.2]
	Aunts/uncles	8,074,278	7,559,242	444,389	66,206	4,441	1.1 [1.1, 1.2]	8,085,069	1.0 [0.9, 1.0]
	Cousins	5,433,963	10,460,326	310,823	93,668	3,112	1.1 [1.1, 1.2]	5,693,457	1.1 [1.0, 1.1]
**Chronic body aches / fatigue**	Within-indiv	2,665,478	2,631,182	10,223	23,336	737	5.2 [4.8, 5.6]	2,749,256	5.3 [4.8, 5.7]
	MZ twins	7,088	NA	NA	NA	NA	NA	7,200	NA
	DZ twins	13,276	NA	NA	NA	NA	NA	13,510	NA
	Full sibs	1,434,189	2,833,942	10,508	23,725	203	2.3 [2.0, 2.7]	1,497,592	2.4 [2.0, 2.8]
	Mothers	2,632,129	2,547,301	61,254	22,436	1,138	2.0 [1.9, 2.1]	2,694,686	1.8 [1.7, 2.0]
	Fathers	2,602,867	2,575,996	3,684	23,141	46	1.4 [1.0, 1.9]	2,645,306	1.2 [0.8, 1.8]
	Mat. half-sibs	243,717	475,427	3,050	8,857	100	1.7 [1.4, 2.1]	259,556	1.8 [1.4, 2.3]

Phenotype	Relative pair	Number of individuals or pairs for odds ratio[a]	Number of individuals unexposed to BPD for odds ratio		Number of individuals exposed to BPD for odds ratio		Adjusted odds ratio[b] [95%CI]	Number of individuals or pairs for hazard ratio[a]	Adjusted hazard ratio[b] [95%CI]
			Control	Case	Control	Case			
	Pat. half-sibs	249,296	487,308	2,912	8,316	56	1.2 [0.9, 1.5]	267,971	1.2 [0.9, 1.7]
	Aunts/uncles	8,074,278	7,922,813	80,818	69,607	1,040	1.4 [1.3, 1.5]	8,085,079	1.3 [1.2, 1.5]
	Cousins	5,433,963	10,724,092	47,057	96,223	557	1.3 [1.2, 1.4]	5,693,457	1.4 [1.2, 1.5]
**Joint pain**	Within-indiv	2,665,478	2,271,721	369,684	19,148	4,925	1.6 [1.5, 1.6]	2,749,256	1.5 [1.4, 1.5]
	MZ twins	7,088	12,129	1,943	89	15	1.0 [0.6, 1.7]	7,200	1.0 [0.5, 2.1]
	DZ twins	13,276	22,793	3,569	166	24	0.9 [0.6, 1.4]	13,510	0.6 [0.3, 1.3]
	Full sibs	1,434,189	2,454,608	389,842	20,403	3,525	1.1 [1.0, 1.1]	1,497,592	1.0 [1.0, 1.1]
	Mothers	2,632,129	1,827,638	780,917	15,578	7,996	1.2 [1.2, 1.2]	2,694,672	1.1 [1.0, 1.1]
	Fathers	2,602,867	1,873,892	705,788	16,701	6,486	1.0 [1.0, 1.1]	2,645,263	0.9 [0.9, 1.0]
	Mat. half-sibs	243,717	410,649	67,828	7,611	1,346	1.1 [1.0, 1.1]	259,556	1.0 [0.9, 1.1]
	Pat. half-sibs	249,296	418,717	71,503	7,142	1,230	1.0 [1.0, 1.1]	267,971	0.9 [0.8, 1.0]
	Aunts/uncles	8,074,278	5,794,051	2,209,580	50,672	19,975	1.0 [1.0, 1.1]	8,084,972	0.9 [0.9, 1.0]
	Cousins	5,433,963	9,248,448	1,522,701	82,935	13,845	1.0 [1.0, 1.0]	5,693,457	0.9 [0.9, 1.0]

95% CI = 95% Wald-type confidence interval. NA = not available (indicates that there were too few observations to estimate the association). Within-indiv = within-individual association. MZ twins = monozygotic twins. DZ twins = dizygotic twins. Mat. = maternal. Pat. = paternal. Sibs = siblings.
[a] Number of unique individuals (for within-individual analyses) or number of unique ways of combining pairs (for relative pair analyses, *i.e.*, a pair may be included twice, first with A as outcome individual and B as exposure individual, then with B as outcome individual and A as exposure individual).
[b] Adjusted for sex, sex of relative, birth year, and birth year of relative where applicable.

Supplementary Table S8. Familial co-aggregation results for behavioral / injury phenotypes with borderline personality disorder

Phenotype	Relative pair	Number of individuals or pairs for odds ratio[a]	Number of individuals unexposed to BPD for odds ratio		Number of individuals exposed to BPD for odds ratio		Adjusted odds ratio[b] [95%CI]	Number of individuals or pairs for hazard ratio[a]	Adjusted hazard ratio[b] [95%CI]
			Controls	Cases	Controls	Cases			
**Accidental falls**	Within-indiv	2,665,478	1,742,661	898,744	13,357	10,716	1.9 [1.8, 1.9]	2,721,304	1.9 [1.8, 2.0]
	MZ twins	7,088	9,457	4,615	55	49	2.3 [1.5, 3.4]	7,194	2.4 [1.5, 3.9]
	DZ twins	13,276	17,012	9,350	114	76	1.3 [0.9, 1.7]	13,501	1.8 [1.2, 2.7]
	Full sibs	1,434,189	1,881,509	962,941	15,281	8,647	1.1 [1.1, 1.2]	1,496,948	1.0 [1.0, 1.1]
	Mothers	2,632,129	1,778,071	830,484	14,891	8,683	1.2 [1.2, 1.3]	2,636,402	1.1 [1.1, 1.2]
	Fathers	2,602,867	1,766,282	813,398	15,101	8,086	1.2 [1.1, 1.2]	2,536,003	1.1 [1.0, 1.1]
	Mat. half-sibs	243,717	298,679	179,798	5,497	3,460	1.1 [1.0, 1.1]	259,436	0.9 [0.8, 0.9]
	Pat. half-sibs	249,296	311,039	179,181	5,192	3,180	1.1 [1.0, 1.1]	267,889	0.9 [0.9, 1.0]
	Aunts/uncles	8,074,278	5,408,089	2,595,542	46,597	24,050	1.1 [1.1, 1.1]	7,814,829	1.0 [1.0, 1.0]
	Cousins	5,433,963	7,078,509	3,692,640	62,907	33,873	1.0 [1.0, 1.1]	5,691,437	0.9 [0.9, 0.9]
**Accidental poisoning**	Within-indiv	2,665,478	2,591,702	49,703	21,674	2,399	6.3 [6.0, 6.6]	2,739,336	13.1 [12.3, 13.9]
	MZ twins	7,088	13,804	268	94	10	5.9 [2.9, 12.0]	7,181	19.9 [7.7, 51.2]
	DZ twins	13,276	25,908	454	182	8	2.3 [1.1, 4.8]	13,501	NA
	Full sibs	1,434,189	2,788,538	55,912	23,057	871	1.9 [1.7, 2.0]	1,497,282	2.5 [2.2, 2.8]
	Mothers	2,632,129	2,577,215	31,340	22,909	665	2.3 [2.1, 2.5]	2,682,561	2.1 [1.7, 2.5]
	Fathers	2,602,867	2,541,564	38,116	22,566	621	1.8 [1.6, 1.9]	2,631,731	1.7 [1.4, 2.1]
	Mat. half-sibs	243,717	463,566	14,911	8,605	352	1.3 [1.2, 1.5]	259,481	1.8 [1.5, 2.2]
	Pat. half-sibs	249,296	476,391	13,829	8,029	343	1.5 [1.3, 1.7]	267,937	2.0 [1.7, 2.5]
	Aunts/uncles	8,074,278	7,887,587	116,044	69,157	1,490	1.5 [1.4, 1.5]	8,040,045	1.3 [1.2, 1.5]
	Cousins	5,433,963	10,551,359	219,790	94,269	2,511	1.3 [1.2, 1.3]	5,692,893	1.4 [1.2, 1.5]
**Transport-related accidents**	Within-indiv	2,665,478	2,262,274	379,131	18,763	5,310	1.8 [1.7, 1.9]	2,738,528	1.7 [1.7, 1.8]
	MZ twins	7,088	12,424	1,648	87	17	1.5 [0.9, 2.6]	7,197	2.0 [1.0, 4.2]
	DZ twins	13,276	23,119	3,243	160	30	1.3 [0.9, 1.9]	13,509	1.5 [0.8, 2.9]
	Full sibs	1,434,189	2,434,680	409,770	19,920	4,008	1.2 [1.1, 1.2]	1,497,392	1.1 [1.0, 1.1]
	Mothers	2,632,129	2,352,105	256,450	20,695	2,879	1.3 [1.2, 1.3]	2,657,512	1.0 [1.0, 1.1]
	Fathers	2,602,867	2,262,362	317,318	19,562	3,625	1.3 [1.3, 1.4]	2,568,649	1.1 [1.0, 1.2]
	Mat. half-sibs	243,717	391,964	86,513	7,189	1,768	1.1 [1.1, 1.2]	259,515	1.0 [0.9, 1.1]
	Pat. half-sibs	249,296	407,121	83,099	6,790	1,582	1.1 [1.1, 1.2]	267,944	1.1 [1.0, 1.2]
	Aunts/uncles	8,074,278	7,108,880	894,751	61,689	8,958	1.1 [1.1, 1.2]	7,896,351	1.0 [1.0, 1.1]
	Cousins	5,433,963	9,138,625	1,632,524	81,142	15,638	1.1 [1.1, 1.1]	5,692,926	1.0 [1.0, 1.0]
**Traumatic brain injury**	Within-indiv	2,665,478	2,380,383	261,022	19,726	4,347	2.3 [2.2, 2.3]	2,723,639	3.3 [3.1, 3.5]
	MZ twins	7,088	12,790	1,282	88	16	2.1 [1.2, 3.6]	7,195	NA

Phenotype	Relative pair	Number of individuals or pairs for odds ratio[a]	Number of individuals unexposed to BPD for odds ratio		Number of individuals exposed to BPD for odds ratio		Adjusted odds ratio[b] [95%CI]	Number of individuals or pairs for hazard ratio[a]	Adjusted hazard ratio[b] [95%CI]
			Controls	Cases	Controls	Cases			
	DZ twins	13,276	23,976	2,386	167	23	1.3 [0.9, 2.0]	13,507	2.3 [1.0, 5.0]
	Full sibs	1,434,189	2,563,210	281,240	20,938	2,990	1.3 [1.2, 1.3]	1,496,884	1.3 [1.2, 1.4]
	Mothers	2,632,129	2,453,616	154,939	21,479	2,095	1.5 [1.5, 1.6]	2,644,685	1.5 [1.3, 1.6]
	Fathers	2,602,867	2,334,104	245,576	20,147	3,040	1.4 [1.4, 1.5]	2,548,934	1.4 [1.2, 1.5]
	Mat. half-sibs	243,717	418,329	60,148	7,739	1,218	1.1 [1.0, 1.2]	259,427	1.0 [0.8, 1.1]
	Pat. half-sibs	249,296	432,359	57,861	7,264	1,108	1.2 [1.1, 1.2]	267,892	1.1 [1.0, 1.3]
	Aunts/uncles	8,074,278	7,336,998	666,633	63,536	7,111	1.2 [1.2, 1.3]	7,842,275	1.1 [1.1, 1.2]
	Cousins	5,433,963	9,657,878	1,113,271	86,020	10,760	1.1 [1.1, 1.1]	5,691,577	1.1 [1.0, 1.1]
**Assault / victimization**	Within-indiv	2,665,478	2,543,351	98,054	19,960	4,113	7.3 [7.0, 7.5]	2,749,057	7.9 [7.5, 8.3]
	MZ twins	7,088	13,676	396	95	9	4.1 [1.9, 8.7]	7,200	NA
	DZ twins	13,276	25,577	785	177	13	2.1 [1.1, 4.0]	13,510	2.2 [0.9, 5.5]
	Full sibs	1,434,189	2,737,420	107,030	22,171	1,757	2.0 [1.9, 2.1]	1,497,586	1.8 [1.7, 2.0]
	Mothers	2,632,129	2,570,715	37,840	22,590	984	2.8 [2.6, 3.0]	2,689,873	2.2 [1.9, 2.5]
	Fathers	2,602,867	2,507,299	72,381	21,849	1,338	2.1 [2.0, 2.2]	2,625,679	1.9 [1.6, 2.2]
	Mat. half-sibs	243,717	444,313	34,164	8,101	856	1.4 [1.3, 1.5]	259,556	1.3 [1.1, 1.4]
	Pat. half-sibs	249,296	458,726	31,494	7,651	721	1.4 [1.3, 1.5]	267,970	1.3 [1.2, 1.5]
	Aunts/uncles	8,074,278	7,834,759	168,872	68,289	2,358	1.6 [1.5, 1.6]	8,044,525	1.5 [1.3, 1.6]
	Cousins	5,433,963	10,356,843	414,306	91,753	5,027	1.4 [1.3, 1.4]	5,693,442	1.3 [1.2, 1.3]
**Self-harm**	Within-indiv	2,665,478	2,539,056	102,349	12,310	11,763	23.1 [22.5, 23.7]	2,748,813	14.8 [14.1, 15.4]
	MZ twins	7,088	13,592	480	75	29	10.5 [6.2, 17.6]	7,200	6.5 [3.1, 13.6]
	DZ twins	13,276	25,425	937	166	24	3.9 [2.4, 6.2]	13,509	4.3 [2.2, 8.2]
	Full sibs	1,434,189	2,726,276	118,174	21,599	2,329	2.5 [2.3, 2.6]	1,497,574	2.3 [2.1, 2.4]
	Mothers	2,632,129	2,497,079	111,476	20,995	2,579	2.6 [2.5, 2.8]	2,669,716	1.8 [1.7, 2.1]
	Fathers	2,602,867	2,483,074	96,606	21,289	1,898	2.2 [2.1, 2.4]	2,626,397	1.5 [1.3, 1.7]
	Mat. half-sibs	243,717	442,714	35,763	7,976	981	1.6 [1.4, 1.7]	259,550	1.5 [1.3, 1.6]
	Pat. half-sibs	249,296	457,287	32,933	7,560	812	1.5 [1.4, 1.6]	267,965	1.4 [1.2, 1.6]
	Aunts/uncles	8,074,278	7,663,995	339,636	65,962	4,685	1.6 [1.5, 1.6]	8,008,208	1.2 [1.1, 1.3]
	Cousins	5,433,963	10,289,895	481,254	90,919	5,861	1.4 [1.3, 1.4]	5,693,437	1.3 [1.2, 1.4]
**Death by suicide**	Within-indiv	2,665,478	2,635,108	6,297	23,437	636	16.9 [15.5, 18.4]	2,749,256	30.3 [27.7, 33.0]
	MZ twins	7,088	NA	NA	NA	NA	NA	7,200	NA
	DZ twins	13,276	NA	NA	NA	NA	NA	13,510	NA
	Full sibs	1,434,189	2,837,748	6,702	23,761	167	2.9 [2.4, 3.4]	1,497,592	2.7 [2.2, 3.4]
	Mothers	2,632,129	2,599,815	8,740	23,331	243	3.0 [2.6, 3.4]	2,694,686	2.8 [2.2, 3.5]
	Fathers	2,602,867	2,557,698	21,982	22,755	432	2.2 [2.0, 2.4]	2,645,306	1.6 [1.3, 2.0]
	Mat. half-sibs	243,717	476,342	2,135	8,882	75	1.9 [1.5, 2.5]	259,556	1.6 [1.1, 2.3]

Phenotype	Relative pair	Number of individuals or pairs for odds ratio[a]	Number of individuals unexposed to BPD for odds ratio		Number of individuals exposed to BPD for odds ratio		Adjusted odds ratio[b] [95%CI]	Number of individuals or pairs for hazard ratio[a]	Adjusted hazard ratio[b] [95%CI]
			Controls	Cases	Controls	Cases			
	Pat. half-sibs	249,296	488,228	1,992	8,330	42	1.3 [0.9, 1.8]	267,971	1.4 [0.9, 2.2]
	Aunts/uncles	8,074,278	7,941,456	62,175	69,813	834	1.5 [1.4, 1.6]	8,085,079	1.5 [1.3, 1.8]
	Cousins	5,433,963	10,743,445	27,704	96,416	364	1.5 [1.3, 1.6]	5,693,457	1.5 [1.3, 1.7]

95% CI = 95% Wald-type confidence interval. NA = not available (Indicates that there were too few observations to estimate the association). Within-indiv = within-individual association. MZ twins = monozygotic twins. DZ twins = dizygotic twins. Mat. = maternal. Pat. = paternal. Sibs = siblings.

[a] Number of unique individuals (for within-individual analyses) or number of unique ways of combining pairs (for relative pair analyses, *i.e.*, a pair may be included twice, first with A as outcome individual and B as exposure individual, then with B as outcome individual and A as exposure individual).

[b] Adjusted for sex, sex of relative, birth year, and birth year of relative where applicable.

Supplementary Table S9. Univariate structural equation modeling results of quantitative genetic analysis

Phenotype	Explained phenotypic variance, percent [95%CI]					
	**Genetic factors (crude A)**	**Genetic factors (adj. A)**	**Common environmental factors (crude C)**	**Common environmental factors (adj. C)**	**Unique environmental factors (crude E)**	**Unique environmental factors (adj. E)**
**Alcohol use disorder**	39% [32%, 45%]	38% [32%, 45%]	3% [-1%, 6%]	3% [-1%, 6%]	59% [55%, 63%]	59% [55%, 63%]
**Drug use disorder**	54% [47%, 61%]	54% [47%, 61%]	6% [3%, 9%]	6% [3%, 9%]	40% [36%, 44%]	40% [36%, 44%]
**Schizophrenia spectrum disorder**	43% [26%, 60%]	42% [25%, 60%]	6% [-2%, 14%]	6% [-3%, 14%]	51% [42%, 60%]	52% [43%, 62%]
**Bipolar disorder**	49% [36%, 62%]	49% [36%, 62%]	3% [-3%, 9%]	3% [-3%, 9%]	48% [41%, 55%]	48% [41%, 55%]
**Depressive disorder**	37% [33%, 42%]	38% [33%, 43%]	4% [2%, 7%]	4% [2%, 7%]	58% [56%, 61%]	58% [55%, 61%]
**Anxiety disorder**	35% [27%, 42%]	35% [27%, 42%]	4% [1%, 8%]	4% [1%, 8%]	62% [57%, 66%]	61%, [57%, 65%]
**Obsessive-compulsive disorder**	50% [33%, 66%]	50% [33%, 67%]	-1% [-9%, 7%]	-1% [-10%, 7%]	52% [43%, 61%]	51% [42%, 60%]
**Acute stress reaction**	29% [20%, 39%]	29% [20%, 39%]	6% [1%, 10%]	5% [1%, 10%]	65% [60%, 70%]	65% [60%, 71%]
**Post-traumatic stress disorder**	34% [19%, 50%]	38% [22%, 54%]	9% [2%, 17%]	10% [2%, 17%]	56% [48%, 65%]	52% [43%, 61%]
**Adjustment disorder**	28% [19%, 37%]	28% [19%, 38%]	3% [-1%, 8%]	3% [-2%, 7%]	68% [63%, 74%]	69% [64%, 74%]
**Anorexia nervosa**	16% [-19%, 51%]	13% [-27%, 53%]	7% [-10%, 24%]	9% [-10%, 29%]	77% [58%, 95%]	78% [56%, 99%]
**Other eating disorder**	44% [25%, 62%]	46% [25%, 67%]	-7% [-16%, 2%]	-7% [-18%, 3%]	64% [54%, 73%]	61% [50%, 73%]
**Borderline personality disorder**	30% [14%, 47%]	35% [17%, 52%]	8% [0%, 15%]	7% [-1%, 15%]	62% [53%, 72%]	58% [48%, 68%]
**Other specific personality disorder**	66% [35%, 96%]	66% [35%, 97%]	-12% [-27%, 3%]	-13% [-28%, 2%]	46% [29%, 63%]	47% [30%, 64%]
**Intellectual disability**	62% [57%, 67%]	53% [48%, 59%]	6% [4%, 9%]	7% [4%, 9%]	32% [29%, 34%]	40% [37%, 43%]
**Autism spectrum disorder**	66% [56%, 76%]	62% [52%, 72%]	6% [1%, 11%]	6% [1%, 11%]	28% [23%, 33%]	32% [27%, 38%]
**Attention deficit hyperactivity disorder**	71% [66%, 77%]	65% [60%, 71%]	5% [2%, 8%]	5% [3%, 8%]	24% [21%, 27%]	30% [26%, 33%]

Phenotype	Explained phenotypic variance, percent [95%CI]					
	**Genetic factors (crude A)**	**Genetic factors (adj. A)**	**Common environmental factors (crude C)**	**Common environmental factors (adj. C)**	**Unique environmental factors (crude E)**	**Unique environmental factors (adj. E)**
**Conduct disorder**	51% [32%, 69%]	50% [32%, 68%]	22% [13%, 31%]	23% [14%, 31%]	27% [17%, 38%]	28% [17%, 38%]
**Childhood anxiety and emotional disorders**	27% [-29%, 83%]	22% [-36%, 81%]	27% [0%, 53%]	27% [0%, 55%]	46% [15%, 77%]	50% [18%, 83%]
**Tic disorder**	48% [14%, 81%]	45% [10%, 80%]	10% [-6%, 26%]	10% [-7%, 26%]	43% [24%, 61%]	46% [27%, 65%]
**Anaphylaxis**	2% [-29%, 33%]	2% [-30%, 33%]	7% [-9%, 22%]	7% [-8%, 22%]	91% [75%, 108%]	92% [75%, 109%]
**Asthma**	45% [40%, 51%]	40% [35%, 46%]	7% [5%, 10%]	7% [5%, 10%]	47% [44%, 50%]	53% [50%, 56%]
**Type 2 diabetes mellitus**	103% [76%, 129%]	101% [74%, 129%]	-16% [-29%, -3%]	-17% [-30%, -3%]	13% [-1%, 27%]	15% [1%, 30%]
**Sleep disorder**	30% [22%, 39%]	30% [22%, 39%]	2% [-2%, 7%]	2% [-2%, 7%]	67% [63%, 72%]	67% [63%, 72%]
**Epilepsy**	25% [10%, 40%]	25% [10%, 40%]	7% [0%, 14%]	7% [0%, 14%]	68% [60%, 76%]	68% [60%, 76%]
**Migraine**	23% [13%, 33%]	24% [14%, 35%]	3% [-2%, 8%]	3% [-2%, 8%]	74% [68%, 79%]	73% [67%, 78%]
**Cardiovascular disease**	25% [17%, 34%]	22% [13%, 31%]	3% [-2%, 7%]	2% [-3%, 6%]	72% [68%, 77%]	77% [72%, 81%]
**Infections**	15% [12%, 18%]	15% [12%, 18%]	5% [3%, 6%]	5% [3%, 6%]	80% [79%, 82%]	80% [79%, 82%]
**Polycystic ovary syndrome[a]**	52% [34%, 70%]	52% [34%, 70%]	5% [-4%, 13%]	5% [-4%, 13%]	43% [34%, 53%]	43% [34%, 53%]
**Sexual pain[a]**	25% [17%, 33%]	25% [17%, 33%]	3% [-1%, 7%]	3% [-1%, 6%]	72% [68%, 76%]	73% [68%, 77%]
**Gastrointestinal pain**	15% [4%, 26%]	14% [3%, 25%]	3% [-2%, 9%]	3% [-2%, 9%]	82% [76%, 88%]	83% [77%, 89%]
**Chronic body aches and fatigue**	62% [32%, 92%]	69% [36%, 102%]	-3% [-18%, 11%]	-6% [-22%, 10%]	42% [26%, 58%]	37% [19%, 55%]
**Accidental poisoning**	29% [18%, 41%]	29% [18%, 41%]	8% [3%, 14%]	8% [2%, 14%]	63% [56%, 69%]	63% [56%, 69%]
**Transport-related accidents**	23% [19%, 27%]	24% [19%, 28%]	4% [2%, 6%]	4% [2%, 6%]	73% [70%, 75%]	72% [70%, 75%]
**Accidental falls**	25% [22%, 28%]	22% [19%, 25%]	4% [2%, 5%]	4% [2%, 5%]	72% [70%, 74%]	74% [73%, 76%]

Phenotype	Explained phenotypic variance, percent [95%CI]					
	Genetic factors (crude A)	Genetic factors (adj. A)	Common environmental factors (crude C)	Common environmental factors (adj. C)	Unique environmental factors (crude E)	Unique environmental factors (adj. E)
**Traumatic brain injury**	12% [7%, 17%]	12% [7%, 17%]	5% [3%, 8%]	5% [3%, 8%]	83% [80%, 86%]	83% [80%, 86%]
**Assault / victimization**	46% [39%, 53%]	48% [41%, 55%]	0% [-3%, 3%]	0% [-4%, 3%]	54% [50%, 58%]	53% [49%, 57%]
**Self-harm**	53% [46%, 59%]	53% [46%, 59%]	2% [-1%, 5%]	2% [-1%, 5%]	45% [42%, 49%]	45% [42%, 49%]
**Death by suicide**	14% [-29%, 57%]	9% [-36%, 54%]	12% [-8%, 32%]	14% [-7%, 35%]	74% [49%, 98%]	77% [52%, 103%]

95%CI = 95% Wald-type confidence interval. Adjusted estimates are adjusted for sex, sex of relative, linear birth year, and linear birth year of relative. Estimates are based on 765,171 unique full sibling, 91,475 maternal half-sibling, and 85,399 paternal half-sibling pairs from independent family clusters. Negative estimates reflect etiological factors that have negative contributions to the phenotypic variance. Structural equation modeling was not performed for congenital hypothyroidism, cystic fibrosis, cerebral palsy, type 1 diabetes mellitus, autoimmune disease, and back, neck, and joint pain, as these phenotypes had a within-individual phenotypic correlation with BPD <0.1 and/or too few observations of discordant and concordant relative pairs.

[a] Female-only analyses were conducted with a reduced dataset of 244,000 full sibling, 35,512 maternal half-sibling, and 35,257 paternal half-sibling female same-sex pairs.

Supplementary Table S10. Bivariate structural equation modeling results of quantitative genetic analysis with borderline personality disorder

Phenotype	Phenotypic correlation[b]	Explained proportion of phenotypic correlation with borderline personality disorder, percent [95%CI]			Genetic correlation[c] (r_g)	Common environmental correlation[c] (r_c)	Unique environmental correlation[c] (r_e)
		Genetic factors (bivariate A)	Common environmental factors (bivariate C)	Unique environmental factors (bivariate E)			
**Alcohol use disorder**	0.51 [0.50, 0.52]	64% [46%, 81%]	0% [-8%, 7%]	36% [25%, 47%]	0.88 [0.59, 1.18]	-0.01 [-0.92, 0.89]	0.32 [0.22, 0.41]
**Drug use disorder**	0.64 [0.63, 0.65]	60% [46%, 75%]	2% [-4%, 8%]	38% [28%, 47%]	0.90 [0.62, 1.18]	0.19 [-0.36, 0.75]	0.50 [0.38, 0.61]
**Schizophrenia spectrum disorder**	0.51 [0.50, 0.53]	56% [27%, 84%]	1% [-11%, 14%]	43% [25%, 61%]	0.78 [0.34, 1.22]	0.09 [-0.78, 0.96]	0.39 [0.23, 0.56]
**Bipolar disorder**	0.62 [0.61, 0.63]	51% [30%, 72%]	5% [-5%, 14%]	45% [31%, 58%]	0.78 [0.46, 1.10]	0.56 [-0.47, 1.59]	0.52 [0.38, 0.66]
**Depressive disorder**	0.69 [0.69, 0.70]	48% [37%, 59%]	4% [0%, 9%]	48% [41%, 55%]	0.92 [0.64, 1.20]	0.56 [0.01, 1.11]	0.57 [0.49, 0.65]
**Anxiety disorder**	0.54 [0.53, 0.55]	55% [38%, 72%]	6% [-1%, 14%]	39% [28%, 50%]	0.86 [0.56, 1.16]	0.60 [-0.10, 1.30]	0.36 [0.26, 0.45]
**Obsessive-compulsive disorder**	0.43 [0.42, 0.45]	51% [18%, 85%]	7% [-8%, 22%]	42% [21%, 63%]	0.56 [0.18, 0.94]	1.00 [-2.57, 4.57]	0.32 [0.16, 0.48]
**Acute stress reaction**	0.54 [0.53, 0.55]	43% [24%, 63%]	11% [2%, 19%]	46% [33%, 59%]	0.75 [0.39, 1.11]	0.87 [0.11, 1.63]	0.40 [0.29, 0.51]
**Post-traumatic stress disorder**	0.58 [0.57, 0.60]	42% [18%, 66%]	14% [4%, 25%]	43% [28%, 59%]	0.90 [0.35, 1.45]	0.69 [0.19, 1.18]	0.43 [0.28, 0.57]
**Adjustment disorder**	0.47 [0.46, 0.48]	61% [39%, 83%]	5% [-4%, 15%]	33% [19%, 47%]	0.97 [0.55, 1.39]	0.52 [-0.41, 1.45]	0.25 [0.14, 0.35]
**Anorexia nervosa**	0.38 [0.36, 0.40]	43% [19%, 66%]	0% (fixed)	57% [34%, 81%]	0.49 [0.19, 0.79]	NA	0.35 [0.20, 0.49]
**Other eating disorder**	0.51 [0.49, 0.52]	50% [19%, 82%]	2% [-12%, 16%]	48% [29%, 68%]	0.78 [0.24, 1.31]	1.00 [-56.61, 58.61]	0.39 [0.23, 0.54]

Phenotype	Phenotypic correlation[b]	Explained proportion of phenotypic correlation with borderline personality disorder, percent [95%CI]			Genetic correlation[c] (r_g)	Common environmental correlation[c] (r_c)	Unique environmental correlation[c] (r_e)
		Genetic factors (bivariate A)	Common environmental factors (bivariate C)	Unique environmental factors (bivariate E)			
**Other specific personality disorder**	0.64 [0.62, 0.65]	55% [42%, 69%]	0% (fixed)	45% [31%, 58%]	1.00 [0.62, 1.38]	NA	0.47 [0.33, 0.61]
**Intellectual disability**	0.55 [0.54, 0.56]	53% [39%, 68%]	6% [0%, 12%]	41% [31%, 50%]	0.69 [0.45, 0.92]	0.45 [-0.02, 0.92]	0.46 [0.36, 0.57]
**Autism spectrum disorder**	0.39 [0.38, 0.41]	58% [28%, 87%]	8% [-4%, 21%]	34% [15%, 53%]	0.48 [0.21, 0.74]	0.56 [-0.35, 1.46]	0.32 [0.14, 0.49]
**Attention deficit hyperactivity disorder**	0.55 [0.54, 0.56]	54% [39%, 69%]	7% [0%, 13%]	39% [29%, 49%]	0.64 [0.42, 0.86]	0.59 [0.02, 1.16]	0.52 [0.39, 0.65]
**Conduct disorder**	0.41 [0.39, 0.43]	58% [16%, 100%]	10% [-7%, 27%]	32% [4%, 60%]	0.57 [0.14, 1.01]	0.32 [-0.25, 0.90]	0.33 [0.04, 0.63]
**Childhood anxiety and emotional disorders**	0.36 [0.32, 0.40]	78% [-8%, 164%]	10% [-27%, 47%]	12% [-44%, 68%]	0.77 [-0.25, 1.79]	0.30 [-0.80, 1.39]	0.09 [-0.32, 0.50]
**Tic disorder**	0.24 [0.20, 0.28]	89% [-8%, 185%]	4% [-37%, 45%]	7% [-57%, 71%]	0.51 [-0.07, 1.09]	0.14 [-1.25, 1.53]	0.03 [-0.28, 0.34]
**Anaphylaxis**	0.11 [0.08, 0.14]	90% [-92%, 272%]	-22% [-104%, 59%]	32% [-82%, 147%]	0.78 [-2.39, 3.95]	-0.40 [-1.98, 1.19]	0.05 [-0.13, 0.22]
**Asthma**	0.13 [0.12, 0.15]	53% [-11%, 116%]	16% [-12%, 43%]	32% [-9%, 73%]	0.19 [-0.04, 0.42]	0.29 [-0.25, 0.84]	0.08 [-0.02, 0.18]
**Type 2 diabetes**	0.21 [0.18, 0.24]	73% [-29%, 176%]	3% [-41%, 47%]	24% [-42%, 89%]	0.33 [-0.14, 0.79]	1.00 [-138.84, 140.84]	0.12 [-0.21, 0.44]
**Sleep disorder**	0.43 [0.41, 0.44]	56% [32%, 79%]	3% [-7%, 13%]	41% [26%, 56%]	0.74 [0.39, 1.08]	0.34 [-0.65, 1.34]	0.28 [0.18, 0.38]
**Epilepsy**	0.19 [0.17, 0.21]	-7% [-80%, 67%]	36% [5%, 68%]	70% [23%, 117%]	-0.04 [-0.53, 0.44]	0.98 [-0.12, 2.07]	0.21 [0.07, 0.36]

Phenotype	Phenotypic correlation[b]	Explained proportion of phenotypic correlation with borderline personality disorder, percent [95%CI]			Genetic correlation[c] (r_g)	Common environmental correlation[c] (r_c)	Unique environmental correlation[c] (r_e)
		Genetic factors (bivariate A)	Common environmental factors (bivariate C)	Unique environmental factors (bivariate E)			
**Migraine**	0.13 [0.11, 0.14]	-18% [-111%, 75%]	44% [3%, 84%]	74% [15%, 133%]	-0.08 [-0.51, 0.35]	1.00 [-0.22, 2.22]	0.14 [0.03, 0.26]
**Cardiovascular disease**	0.15 [0.13, 0.16]	-6% [-79%, 67%]	24% [-8%, 55%]	83% [36%, 129%]	-0.03 [-0.42, 0.36]	0.97 [-0.83, 2.77]	0.18 [0.08, 0.28]
**Infections**	0.21 [0.20, 0.22]	51% [22%, 81%]	7% [-6%, 20%]	42% [23%, 61%]	0.47 [0.18, 0.77]	0.24 [-0.22, 0.69]	0.13 [0.07, 0.18]
**Polycystic ovary syndrome[a]**	0.12 [0.10, 0.15]	31% [-97%, 160%]	3% [-54%, 61%]	65% [-15%, 146%]	0.09 [-0.27, 0.44]	0.08 [-1.22, 1.38]	0.16 [-0.04, 0.37]
**Sexual pain[a]**	0.20 [0.19, 0.21]	74% [28%, 120%]	4% [-17%, 24%]	22% [-8%, 52%]	0.47 [0.16, 0.78]	0.20 [-0.87, 1.27]	0.07 [-0.02, 0.16]
**Gastrointestinal problems**	0.17 [0.15, 0.19]	53% [-16%, 121%]	8% [-22%, 39%]	39% [-5%, 82%]	0.41 [-0.15, 0.98]	0.29 [-0.75, 1.33]	0.09 [-0.01, 0.20]
**Chronic body aches / fatigue**	0.27 [0.25, 0.30]	58% [-25%, 140%]	19% [-16%, 53%]	24% [-30%, 77%]	0.39 [-0.18, 0.97]	1.00 [-2.46, 4.46]	0.12 [-0.15, 0.40]
**Accidental poisoning**	0.32 [0.31, 0.34]	43% [6%, 80%]	7% [-9%, 23%]	50% [26%, 74%]	0.44 [0.05, 0.83]	0.30 [-0.37, 0.97]	0.27 [0.14, 0.40]
**Transport-related accident**	0.13 [0.11, 0.14]	40% [-16%, 97%]	11% [-14%, 35%]	49% [13%, 85%]	0.18 [-0.08, 0.43]	0.25 [-0.34, 0.83]	0.09 [0.02, 0.17]
**Accidental falls**	0.14 [0.13, 0.15]	29% [-14%, 73%]	-1% [-20%, 18%]	71% [44%, 99%]	0.15 [-0.08, 0.38]	-0.02 [-0.55, 0.52]	0.15 [0.09, 0.22]
**Traumatic brain injury**	0.17 [0.16, 0.18]	22% [-25%, 68%]	18% [-3%, 38%]	61% [31%, 90%]	0.18 [-0.21, 0.57]	0.48 [-0.13, 1.09]	0.15 [0.07, 0.22]
**Assault / victimization**	0.38 [0.37, 0.39]	74% [63%, 85%]	0% (fixed)	26% [15%, 37%]	0.68 [0.47, 0.89]	NA	0.18 [0.10, 0.26]
**Self-harm**	0.64 [0.63, 0.65]	54% [40%, 69%]	0% [-6%, 6%]	45% [36%, 55%]	0.80 [0.55, 1.04]	0.01 [-1.06, 1.09]	0.57 [0.46, 0.68]
**Death by suicide**	0.43 [0.40, 0.46]	49% [-8%, 106%]	14% [-11%, 38%]	37% [1%, 73%]	0.99 [-1.08, 3.06]	0.65 [-0.62, 1.92]	0.24 [0.01, 0.47]

95%CI = 95% Wald-type confidence interval. Adjusted estimates are adjusted for sex, sex of relative, linear birth year, and linear birth year of relative. Estimates are based on 765,171 unique full sibling, 91,475 maternal half-sibling, and 85,399 paternal half-sibling pairs from independent family clusters. Negative estimates reflect etiological factors that have negative contributions to the proportion of phenotypic correlation. Structural equation modeling was not performed for congenital hypothyroidism, cystic fibrosis, cerebral palsy, type 1 diabetes mellitus, autoimmune disease, and back, neck, and joint pain, as these phenotypes had a within-individual phenotypic correlation with BPD <0.1 and/or too few observations of discordant and concordant relative pairs.

[a] Female-only analyses were conducted with a reduced dataset of 244,000 full sibling, 35,512 maternal half-sibling, and 35,257 paternal half-sibling female same-sex pairs.

[b] Phenotypic association calculated with tetrachoric correlation.

[c] Genetic, common environmental, and unique environmental correlations derived from the bivariate estimate of contribution of shared additive genetic, common environmental, and unique environmental factors to the observed phenotypic correlation between phenotypes.

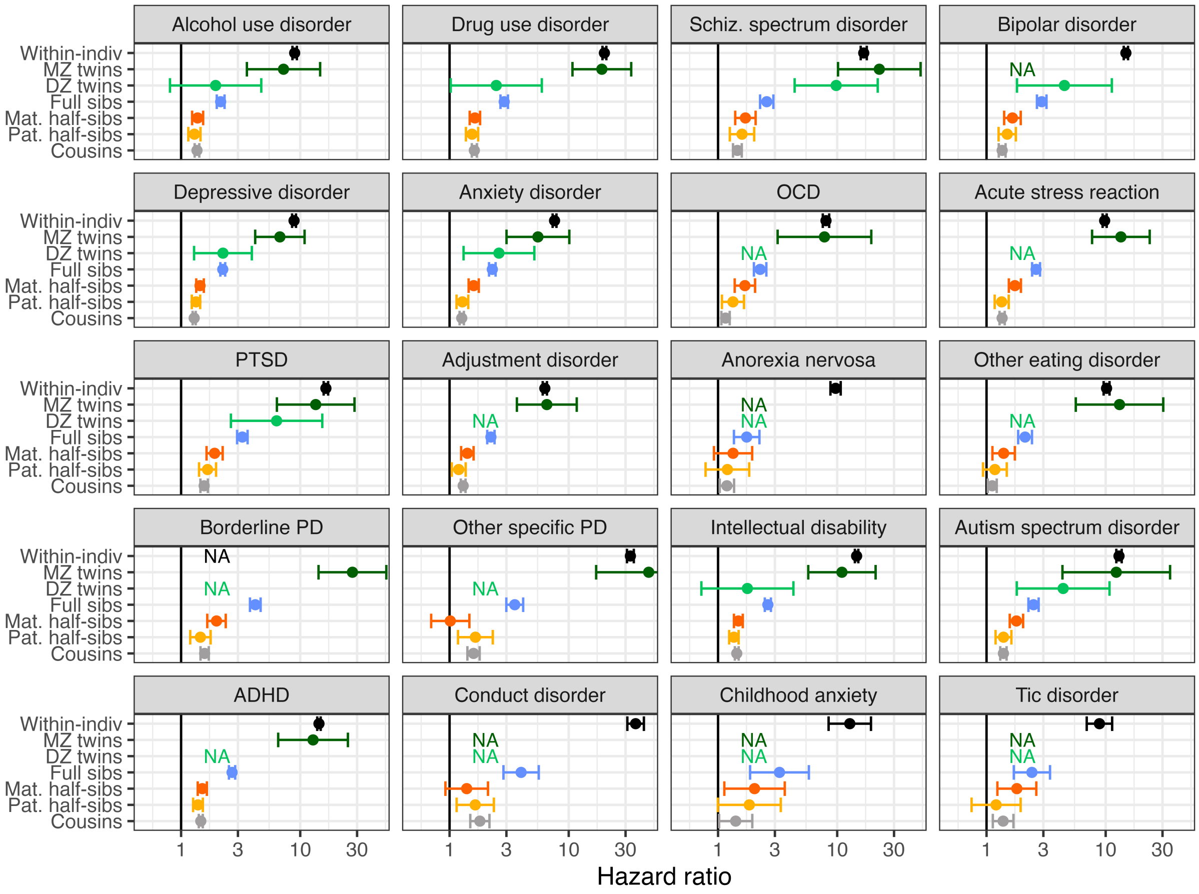

Supplementary Figure S2. Hazard ratio of psychiatric phenotype diagnosis when having a borderline personality disorder diagnosis oneself, or a relative diagnosed with borderline personality disorder

Note. Hazard ratios are adjusted for sex, sex of relative, birth year, and birth year of relative where applicable and shown with 95% Wald-type confidence intervals based on cluster-robust standard errors. X-axis is log-transformed and scaled to fit the minimum and maximum point estimates observed for all phenotypes studied. NA = not available (indicates that there were too few observations to estimate the association). Within-indiv = within-individual association. MZ twins = monozygotic twins. DZ twins = dizygotic twins. Mat. = maternal. Pat. = paternal. Sibs = siblings. OCD = obsessive compulsive disorder. PTSD = post- traumatic stress disorder. PD = personality disorder. ADHD = attention deficit hyperactivity disorder.

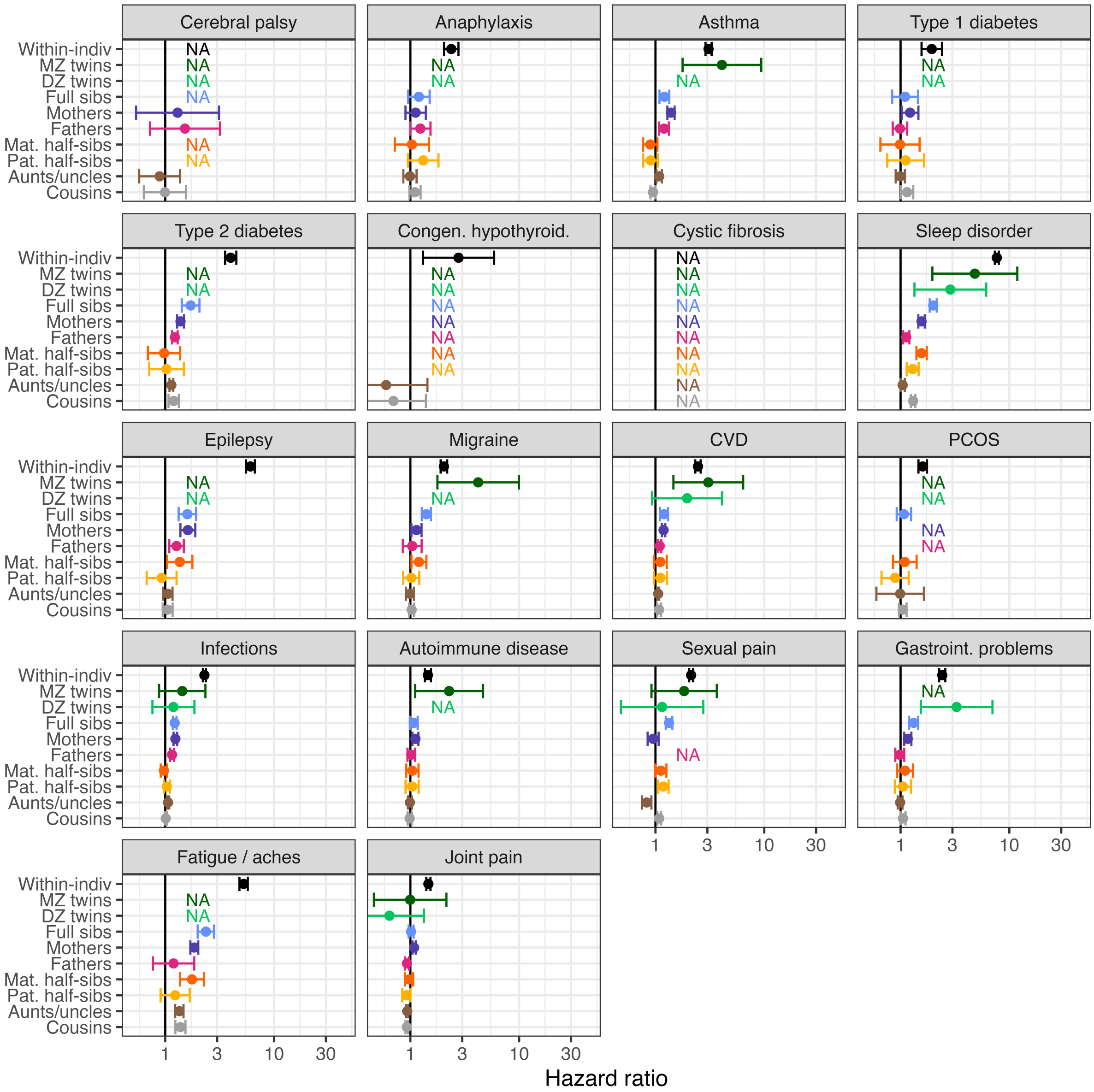

Supplementary Figure S3. Hazard ratio of somatic phenotype diagnosis when having a borderline personality disorder diagnosis oneself, or a relative diagnosed with borderline personality disorder

Note. Hazard ratios are adjusted for sex, sex of relative, birth year, and birth year of relative where applicable and shown with 95% Wald-type confidence intervals based on cluster-robust standard errors. X-axis is log-transformed and scaled to fit the minimum and maximum point estimates observed for all phenotypes studied. NA = not available (indicates that there were too few observations to estimate the association). Within-indiv = within-individual association. MZ twins = monozygotic twins. DZ twins = dizygotic twins. Mat. = maternal. Pat. = paternal. Sibs = siblings. CVD = cardiovascular disease. PCOS = polycystic ovary syndrome.

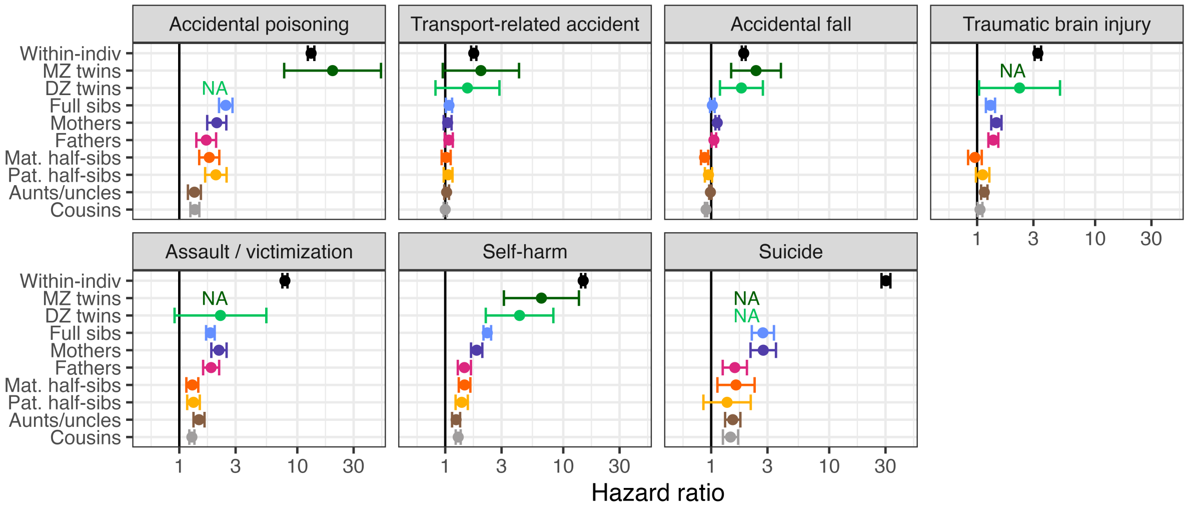

Supplementary Figure S4. Hazard ratio of behavioral/injury phenotype diagnosis when having a borderline personality disorder diagnosis oneself, or a relative diagnosed with borderline personality disorder

Note. Hazard ratios are adjusted for sex, sex of relative, birth year, and birth year of relative where applicable and shown with 95% Wald-type confidence intervals based on cluster-robust standard errors. X-axis is log-transformed and scaled to fit the minimum and maximum point estimates observed for all phenotypes studied. NA = not available (indicates that there were too few observations to estimate the association). Within-indiv = within-individual association. MZ twins = monozygotic twins. DZ twins = dizygotic twins. Mat. = maternal. Pat. = paternal. Sibs = siblings.

Supplementary References

1. Källén B, Källén K. The Swedish Medical Birth Register - a summary of content and quality. Vols. 2003-112–3. Socialstyrelsen; 2003.

2. World Health Organization (WHO). International Statistical Classification of Diseases, Injuries and Causes of Death, 8th Revision (ICD-8). Geneva, Switzerland; 1965.

3. World Health Organization (WHO). International Classification of Diseases, Injuries and Causes of Death, Ninth Revision (ICD-9). Geneva, Switzerland; 1987.

4. World Health Organization (WHO). International Statistical Classification of Diseases and Related Health Problems, Tenth Revision, Swedish Version (ICD-10-SE). Geneva, Switzerland: Swedish National Board of Health and Welfare; 1996.

5. Magnusson PKE, Almqvist C, Rahman I, Ganna A, Viktorin A, Walum H, et al. The Swedish Twin Registry: Establishment of a Biobank and Other Recent Developments. Twin Res Hum Genet. 2013 Feb;16(1):317–29.

6. Ludvigsson JF, Andersson E, Ekbom A, Feychting M, Kim JL, Reuterwall C, et al. External review and validation of the Swedish national inpatient register. BMC Public Health. 2011 June 9;11(1):450.

7. Forsberg L, Rydh H, Björkenstam E, Jacobsson A, Nyqvist K, Heurgren M. Kvalitet och innehåll i patientregistret. Utskrivningar från slutenvården 1964-2007 och besök i specialiserad öppenvård (exklusive primärvårdsbesök) 1997-2007. (Quality and content of the Patient Register) [Internet]. Vol. Artikelnr 2009-125-15. Stockholm: Socialstyrelsen; 2009. Available from: https://www.socialstyrelsen.se/contentassets/12ab370b4cec441db1b8b0c78b9d6a1d/2009-125-15_200912515_rev2.pdf

8. Solmi M, Radua J, Olivola M, Croce E, Soardo L, Salazar de Pablo G, et al. Age at onset of mental disorders worldwide: large-scale meta-analysis of 192 epidemiological studies. Mol Psychiatry. 2022 Jan;27(1):281–95.

9. Porta M, Curletto G, Cipullo D, Rigault de la Longrais R, Trento M, Passera P, et al. Estimating the delay between onset and diagnosis of type 2 diabetes from the time course of retinopathy prevalence. Diabetes Care. 2014 June;37(6):1668–74.

10. Zetterqvist J, Sjölander A. Doubly Robust Estimation with the R Package drgee. Epidemiol Methods. 2015 Dec 1;4(1):69–86.

11. R Core Team. R: A Language and Environment for Statistical Computing [Internet]. Vienna, Austria: R Foundation for Statistical Computing; 2023. Available from: https://www.R-project.org/

12. Revelle W. psych: Procedures for Psychological, Psychometric, and Personality Research [Internet]. Evanston, Illinois: Northwestern University; 2024. Available from: https://CRAN.R-project.org/package=psych

13. Knopik VS, Neiderhiser JM, DeFries JC, Plomin R. Behavioral genetics. Seventh edition. New York: Worth Publishers, Macmillan Learning; 2017. 508 p.

14. Sullivan PF, Eaves LJ. Evaluation of analyses of univariate discrete twin data. Behav Genet. 2002 May;32(3):221–7.

15. Yilmaz Z, Hardaway JA, Bulik CM. Genetics and Epigenetics of Eating Disorders. Adv Genomics Genet. 2015;5:131–50.

16. Skoglund C, Tiger A, Rück C, Petrovic P, Asherson P, Hellner C, et al. Familial risk and heritability of diagnosed borderline personality disorder: a register study of the Swedish population. Mol Psychiatry. 2021 Mar;26(3):999–1008.

17. Therneau TM, Grambsch PM. Modeling Survival Data: Extending the Cox Model. New York: Springer; 2000.

18. Neale MC, Hunter MD, Pritikin JN, Zahery M, Brick TR, Kirkpatrick RM, et al. OpenMx 2.0: Extended Structural Equation and Statistical Modeling. Psychometrika. 2016 June 1;81(2):535–49.
